# Supplementary material for: Deciphering synergetic core-shell transformation from [Mo6O22@Ag44] to [Mo8O28@Ag50]
Source: Nat Commun. 2018 Oct 23;9:4407. doi: 10.1038/s41467-018-06755-4 (PMC6199286; doi:10.1038/s41467-018-06755-4)
Supplement: Supplementary file 1 — Supporting information [file 41467_2018_6755_MOESM1_ESM.pdf]

Supplementary Information

Deciphering Synergetic Core-Shell Transformation from [Mo_6O_{22}@Ag_{44}] to [Mo_8O_{28}@Ag_{50}]

Zhi Wang,[1†] Hai-Feng Su,[2†] Chen-Ho Tung,[1] Di Sun[*,1] and Lan-Sun Zheng[2]

[1]Key Laboratory of the Colloid and Interface Chemistry, Ministry of Education, and School of Chemistry and Chemical Engineering, Shandong University, Jinan, 250100, P. R. China.

[2]State Key Laboratory for Physical Chemistry of Solid Surfaces and Department of Chemistry, College of Chemistry and Chemical Engineering, Xiamen University, Xiamen, 361005, P. R. China.

†These authors contributed equally to this work.

*To whom correspondence should be addressed. E-mail: dsun@sdu.edu.cn.

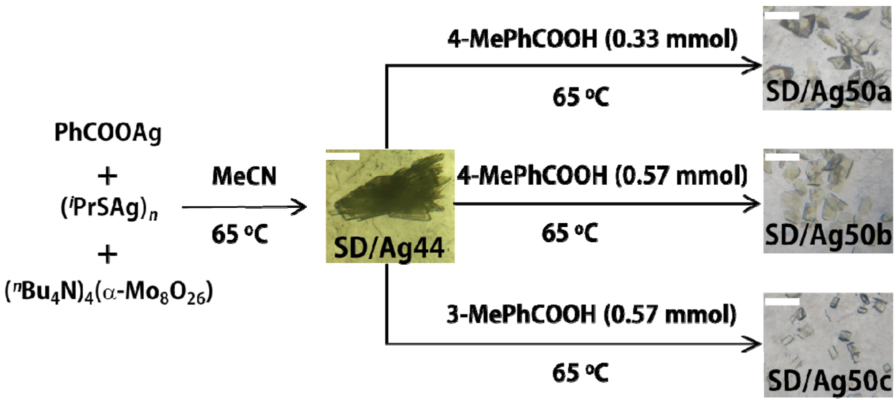

Supplementary Figure 1: Synthesis routes for **SD/Ag50a-SD/Ag50c**. The scale bar is 1 mm

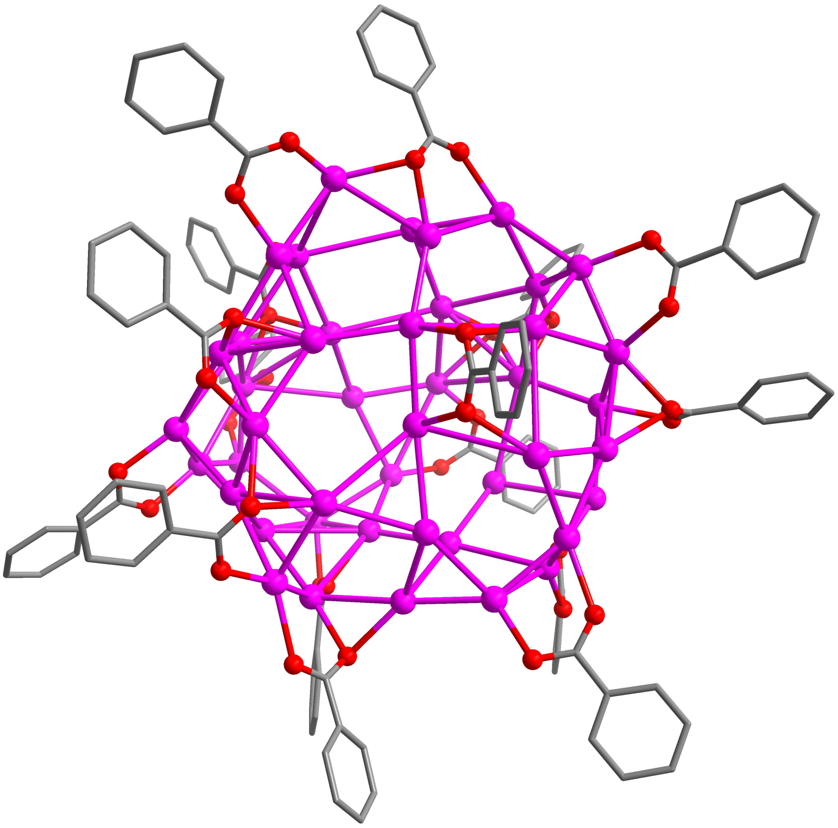

Supplementary Figure 2: The $PhCOO^-$ ligands coordinated on the silver shell of **SD/Ag44**.

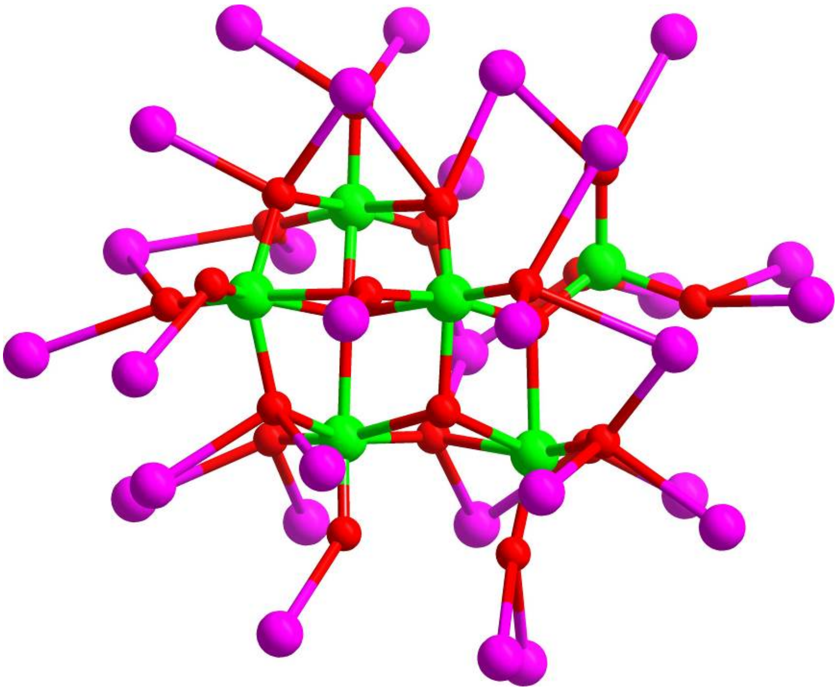

Supplementary Figure 3: The binding mode of $[Mo_6O_{22}]^{8-}$ towards silver atoms in **SD/Ag44**.

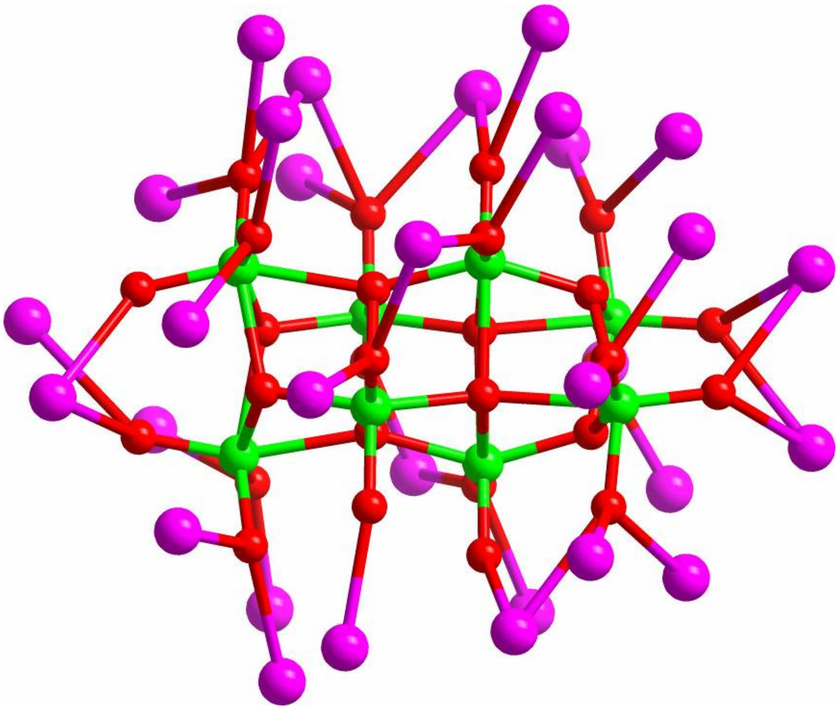

Supplementary Figure 4: The binding mode of $[Mo_8O_{28}]^{8-}$ towards silver atoms in **SD/Ag50**.

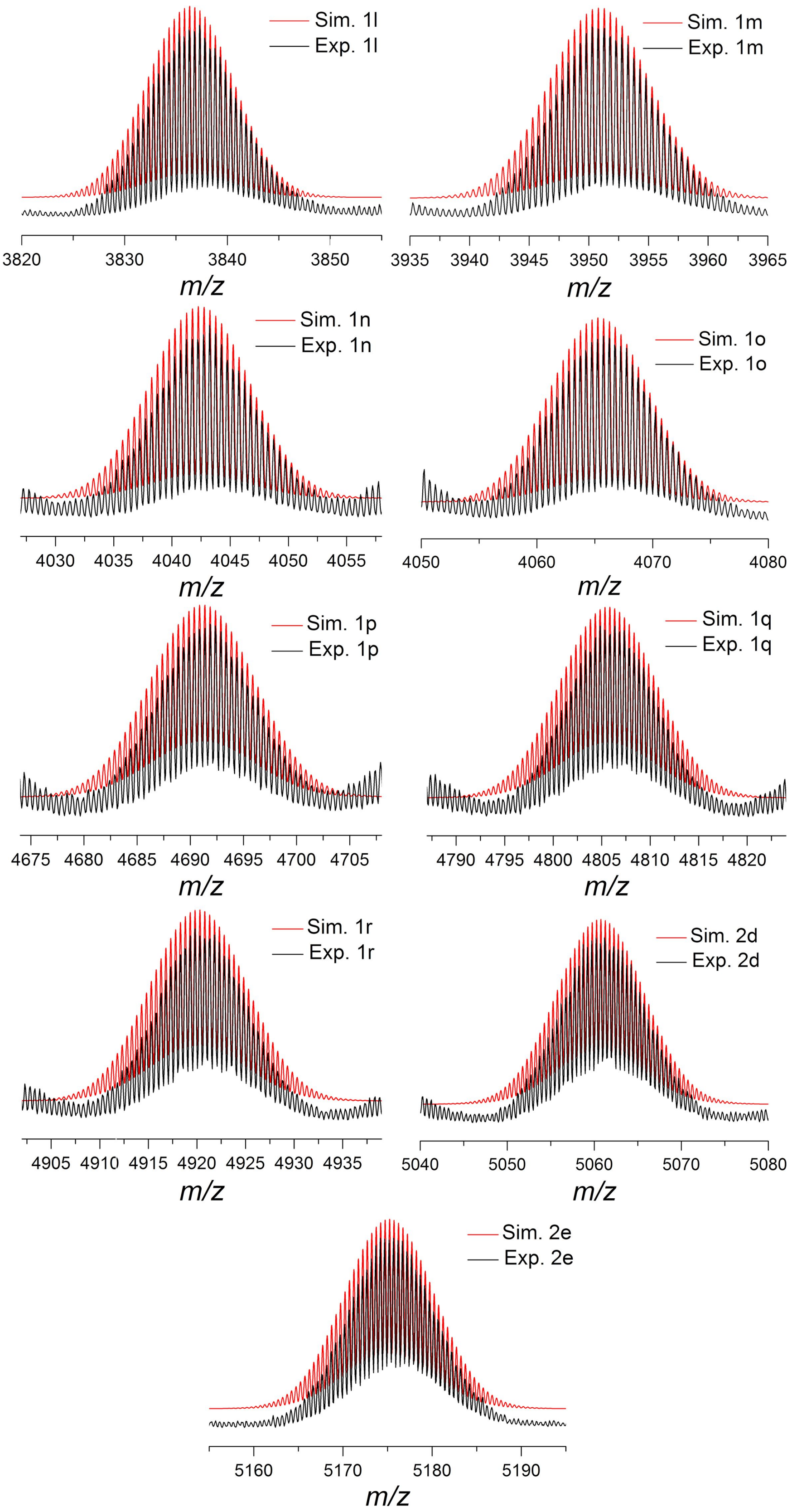

Supplementary Figure 5: The theoretical and experimental isotope distributions of species **1l-1r, 2d** and **2e**.

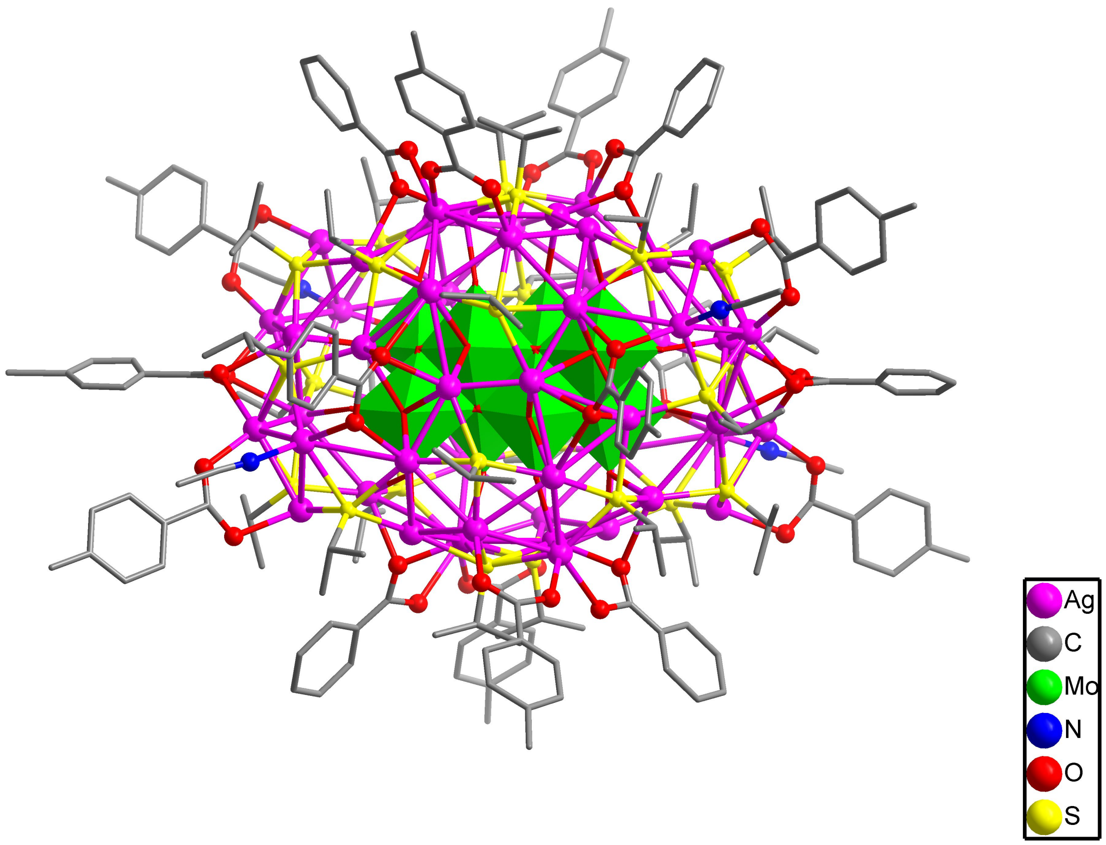

Supplementary Figure 6: The X-ray structure of **SD/Ag50a**.

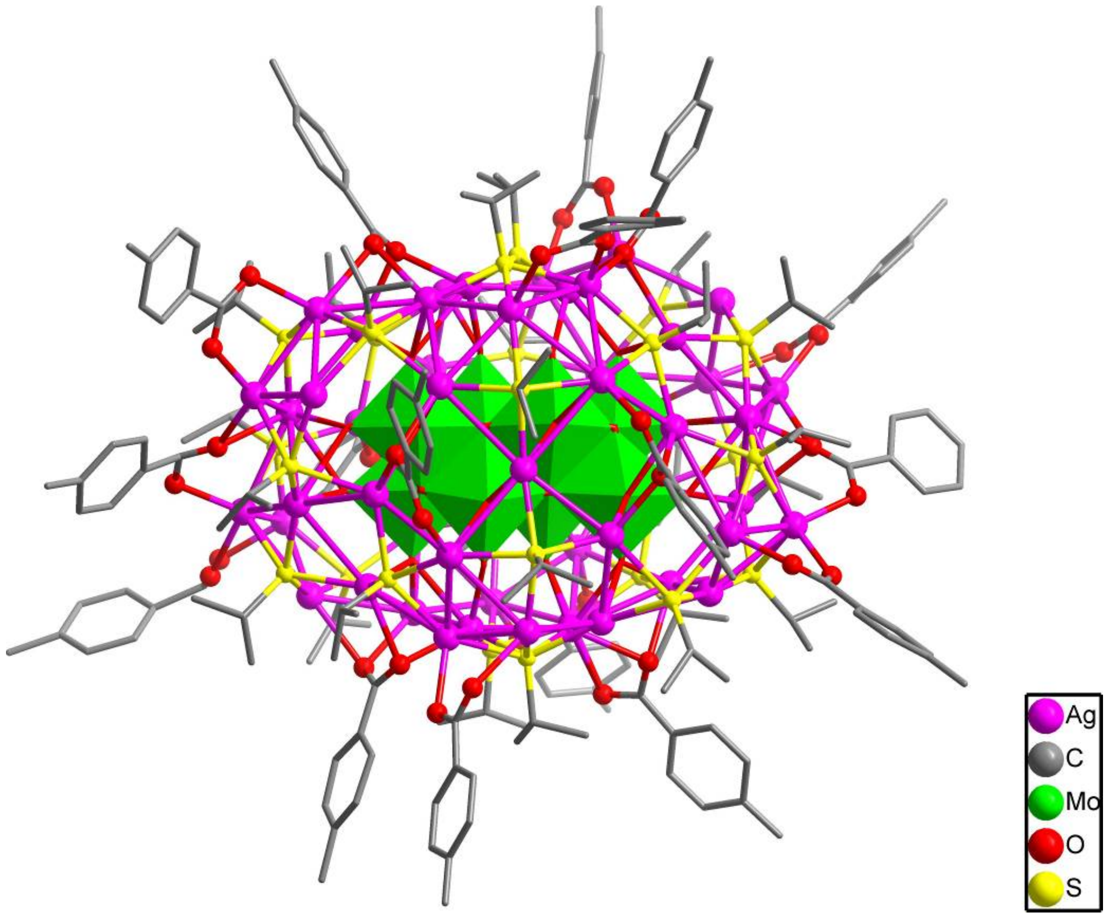

Supplementary Figure 7: The X-ray structure of **SD/Ag50b**.

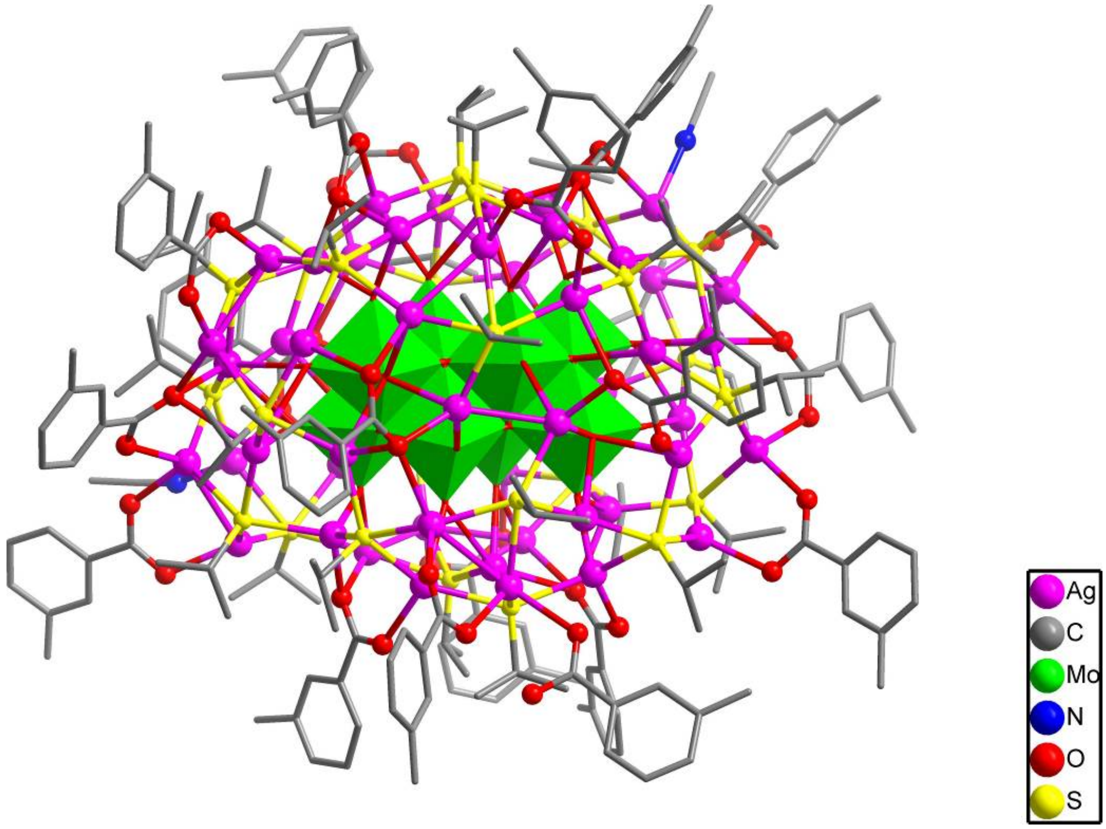

Supplementary Figure 8: The X-ray structure of **SD/Ag50c**.

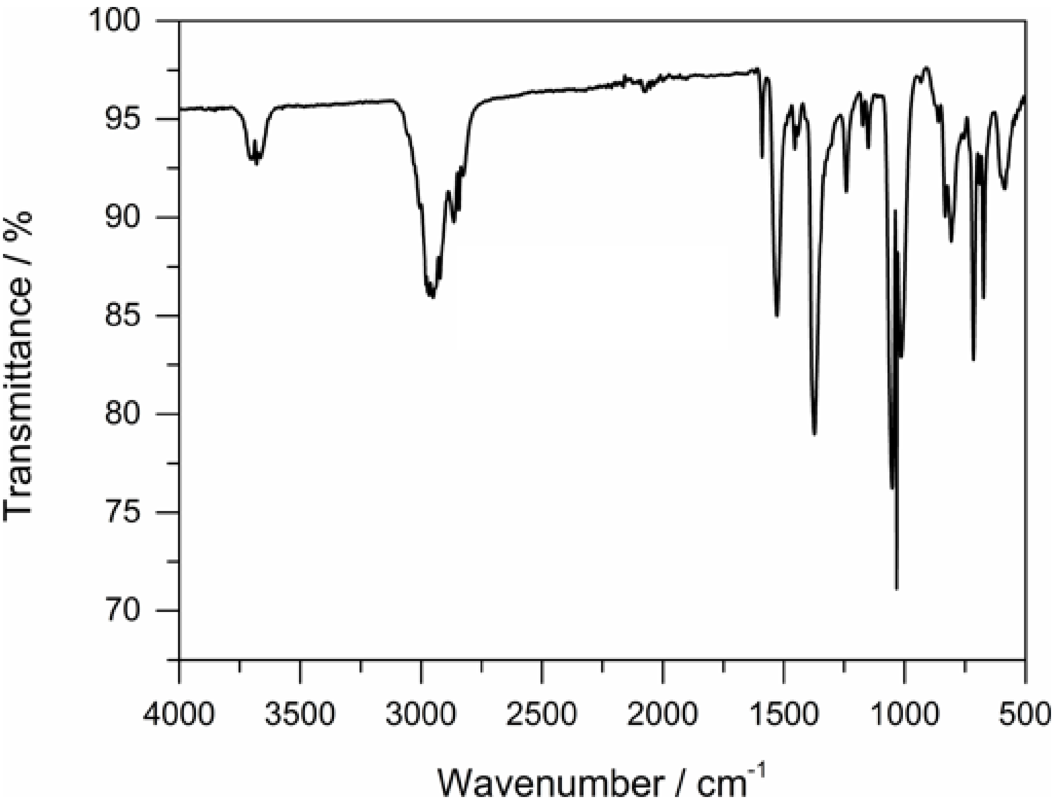

Supplementary Figure 9: The IR of **SD/Ag44**.

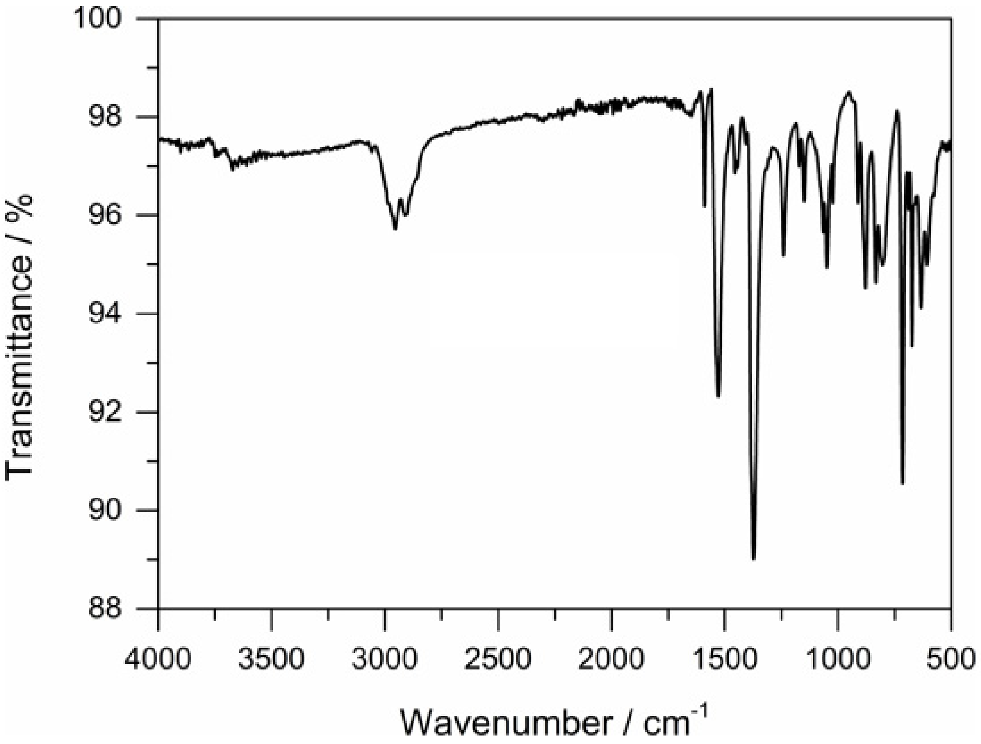

Supplementary Figure 10: The IR of **SD/Ag50**.

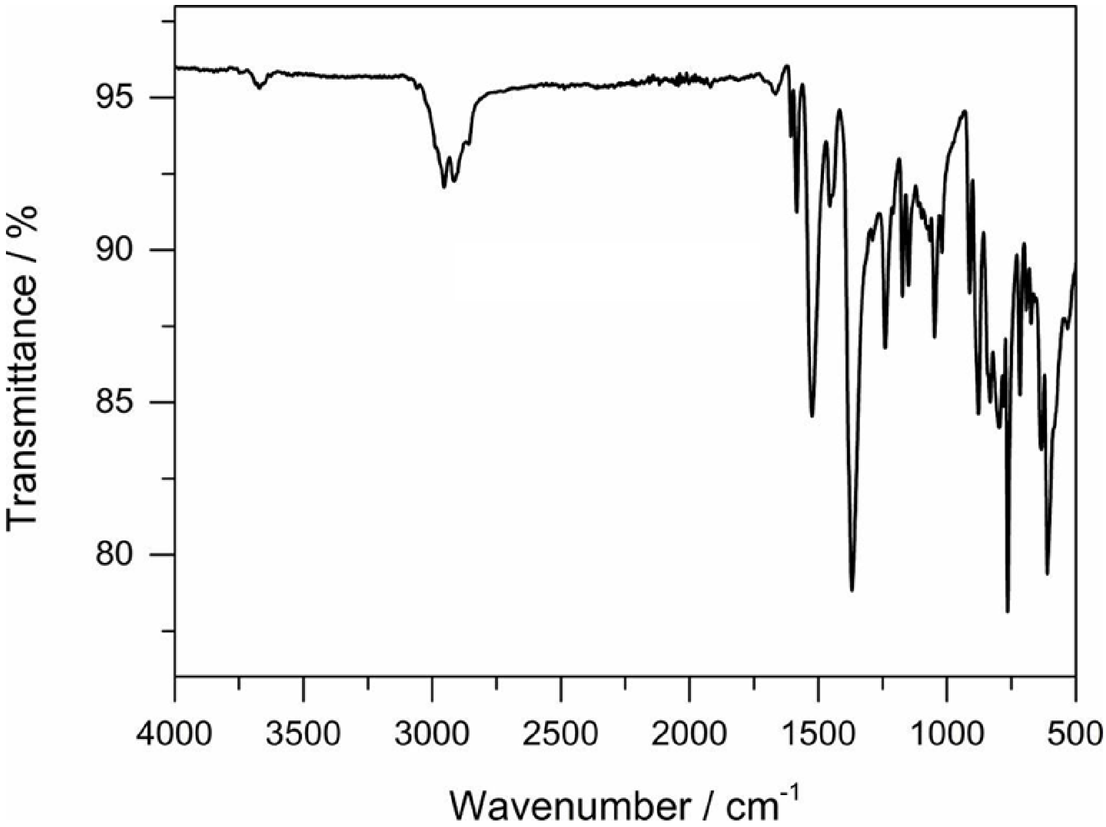

Supplementary Figure 11: The IR of **SD/Ag50a**.

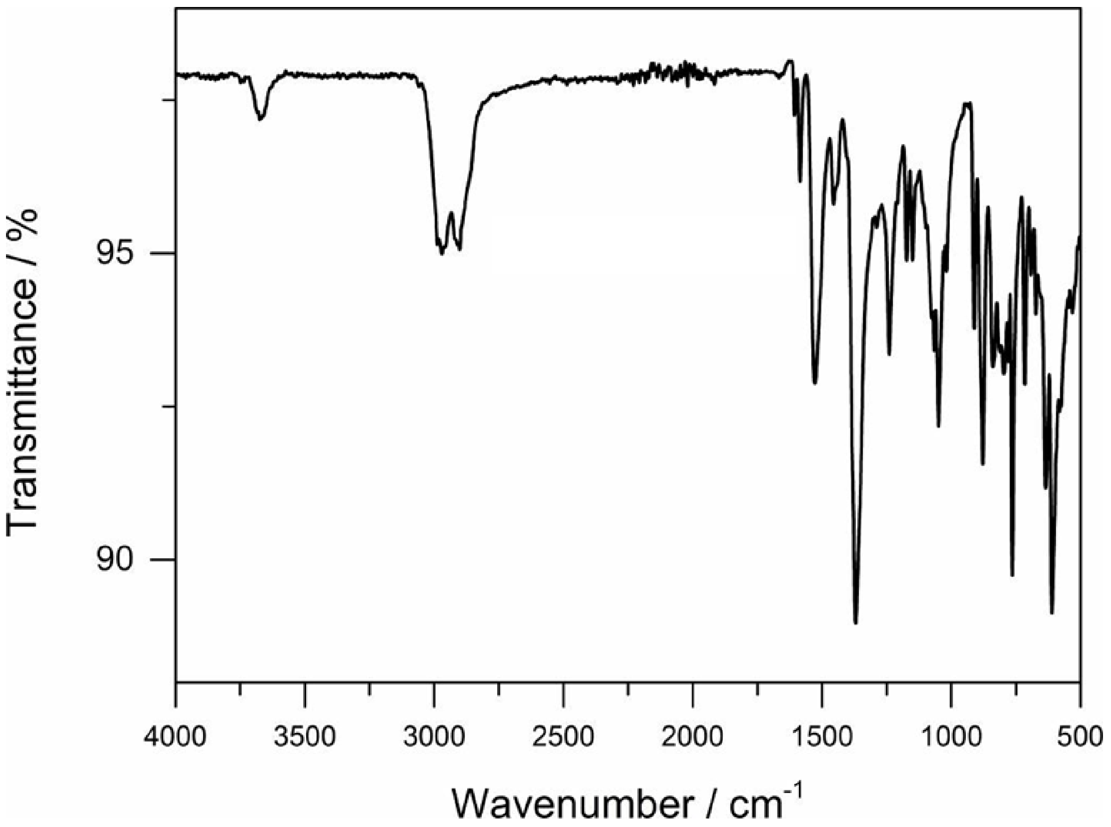

Supplementary Figure 12: The IR of **SD/Ag50b**.

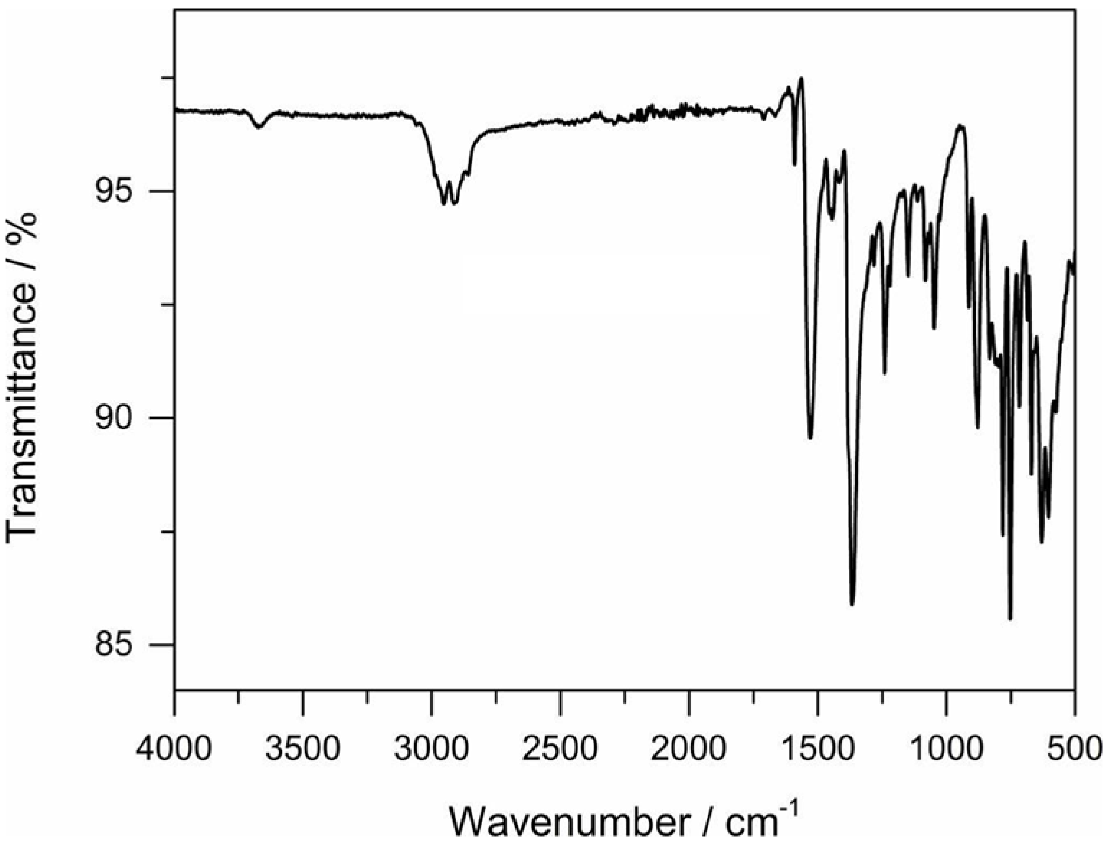

Supplementary Figure 13: The IR of **SD/Ag50c**.

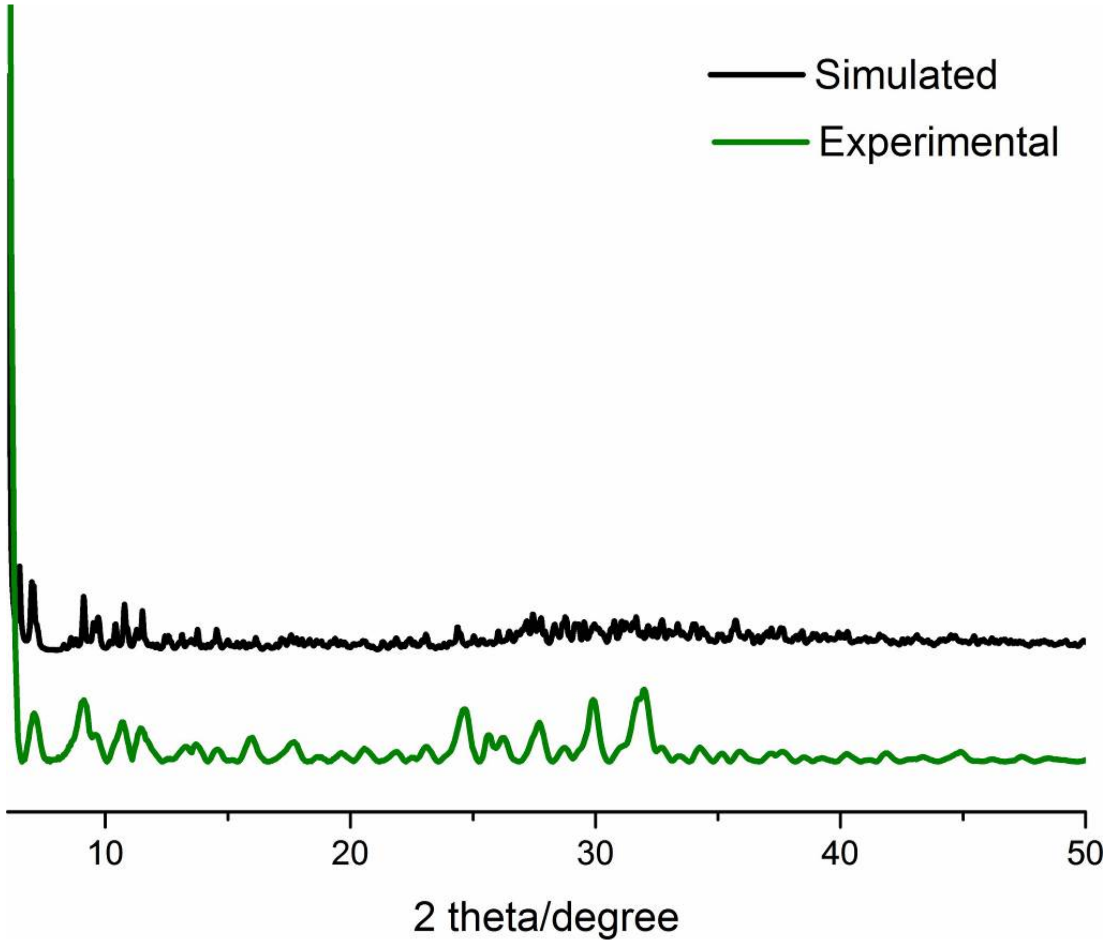

Supplementary Figure 14: Compared PXRD patterns of **SD/Ag44**.

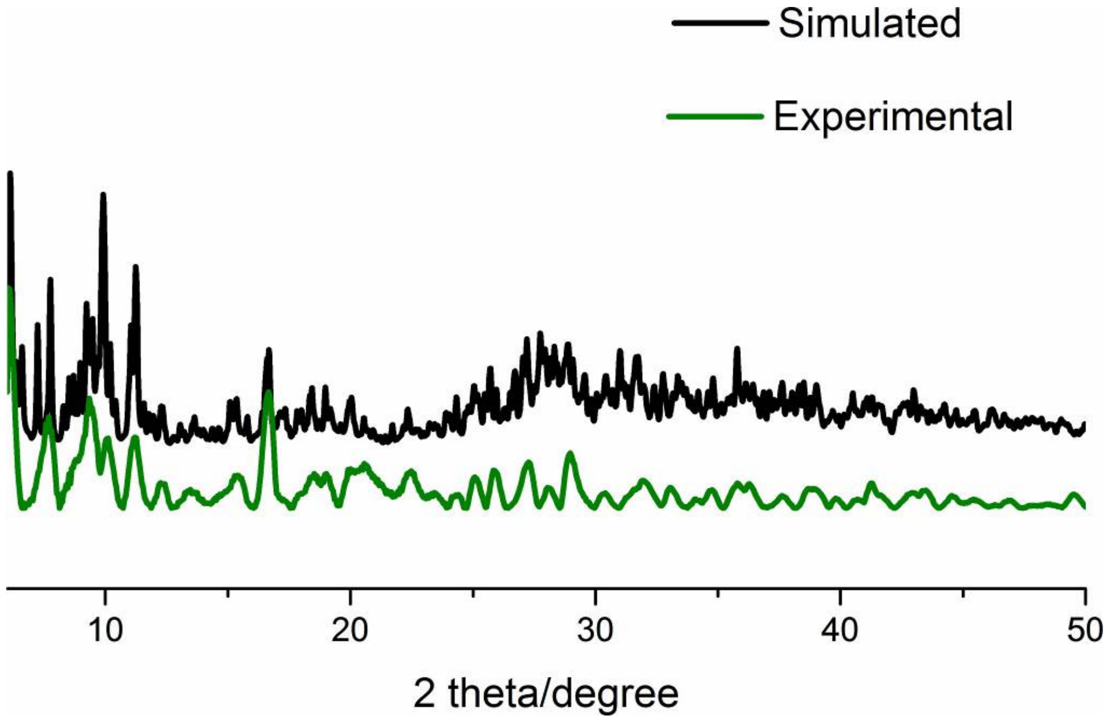

Supplementary Figure 15: Compared PXRD patterns of **SD/Ag50**.

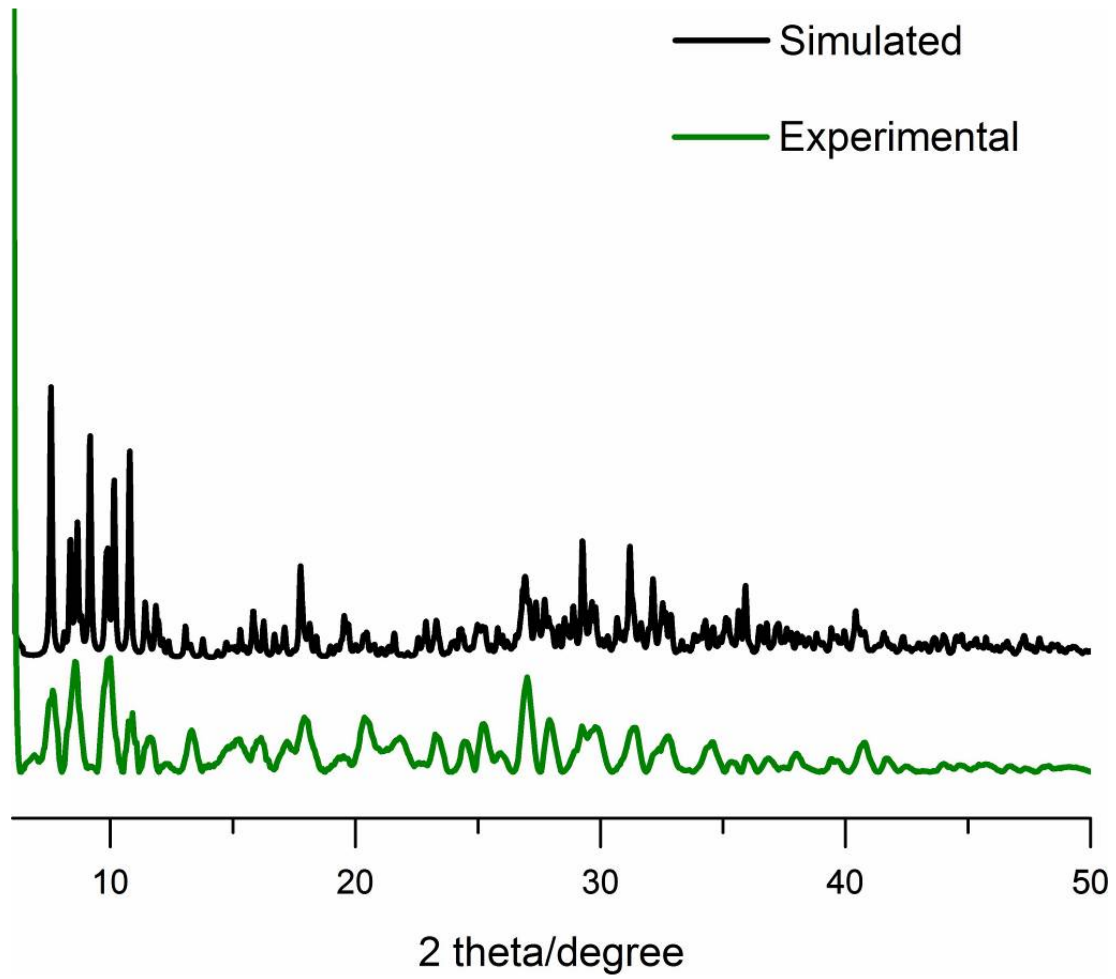

Supplementary Figure 16: Compared PXRD patterns of **SD/Ag50a**.

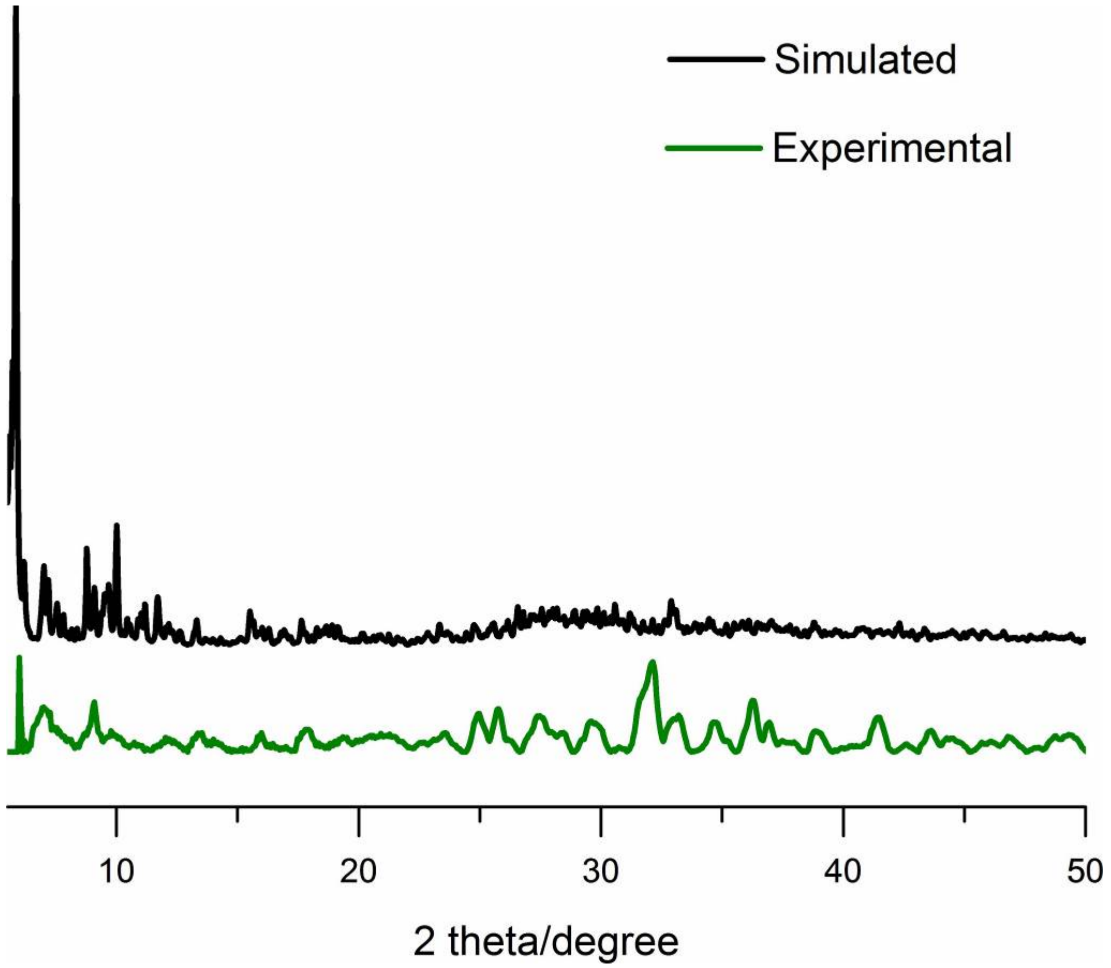

Supplementary Figure 17: Compared PXRD patterns of **SD/Ag50b**.

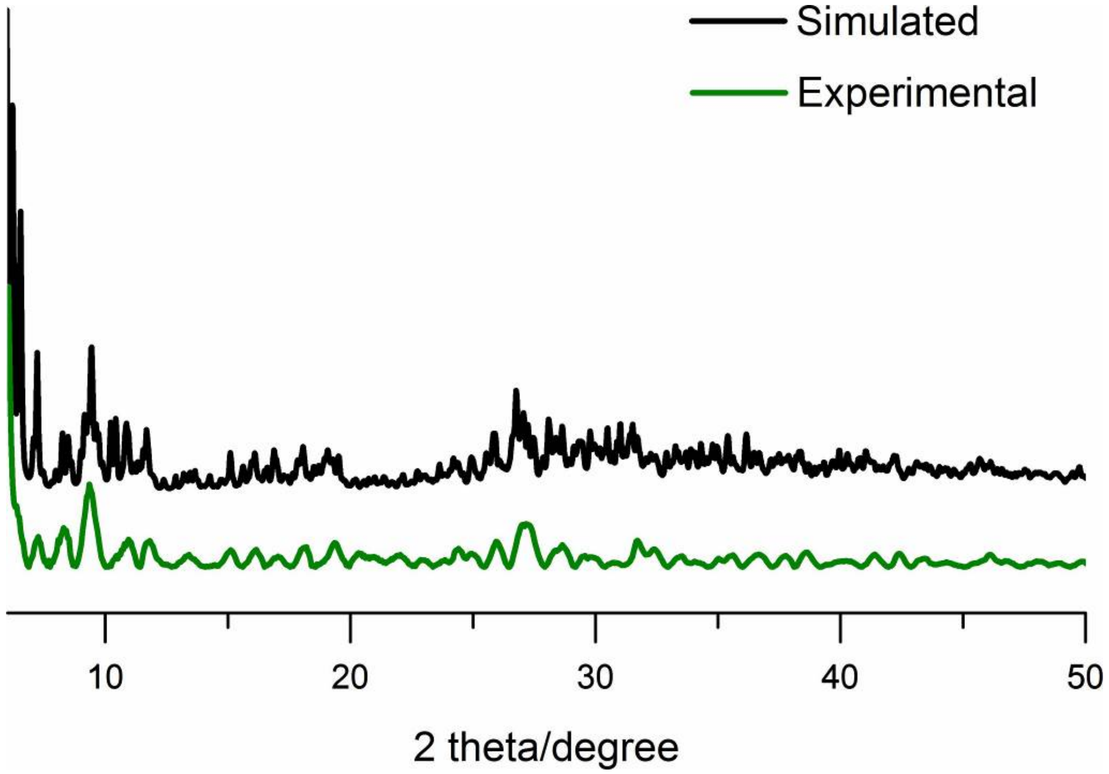

Supplementary Figure 18: Compared PXRD patterns of **SD/Ag50c**.

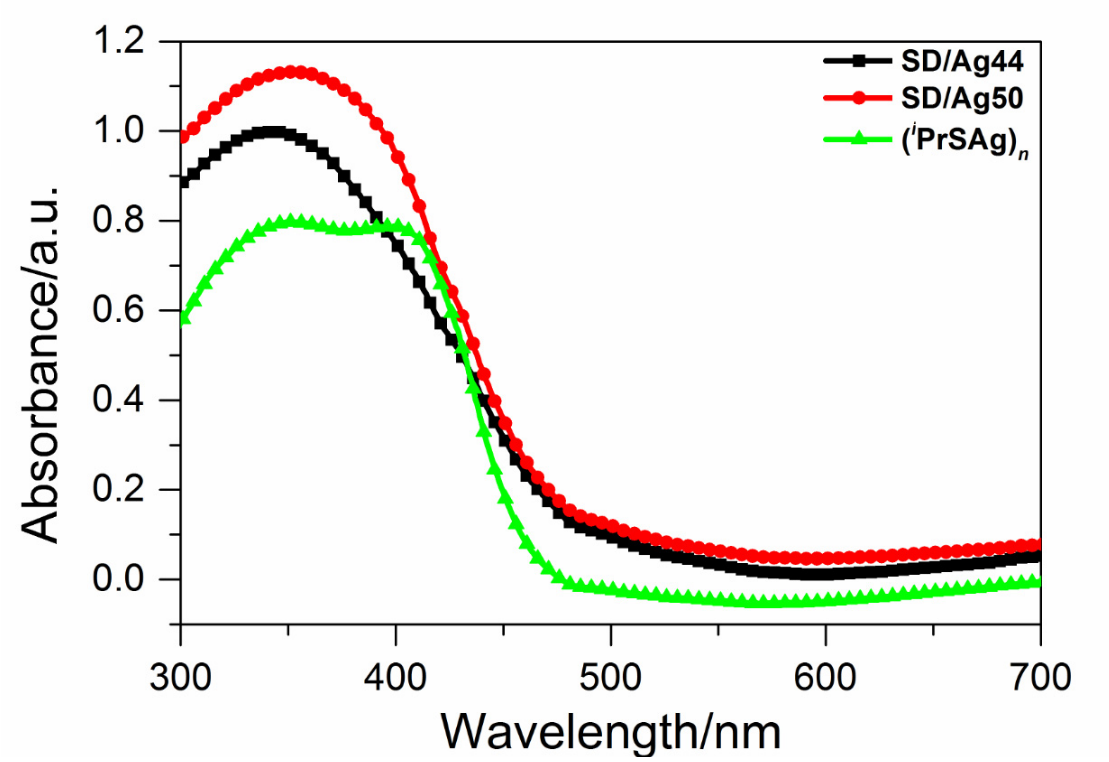

Supplementary Figure 19: The UV/Vis diffuse reflectance spectra of **SD/Ag44, SD/Ag50** and (^{i}PrSAg)$_n$.

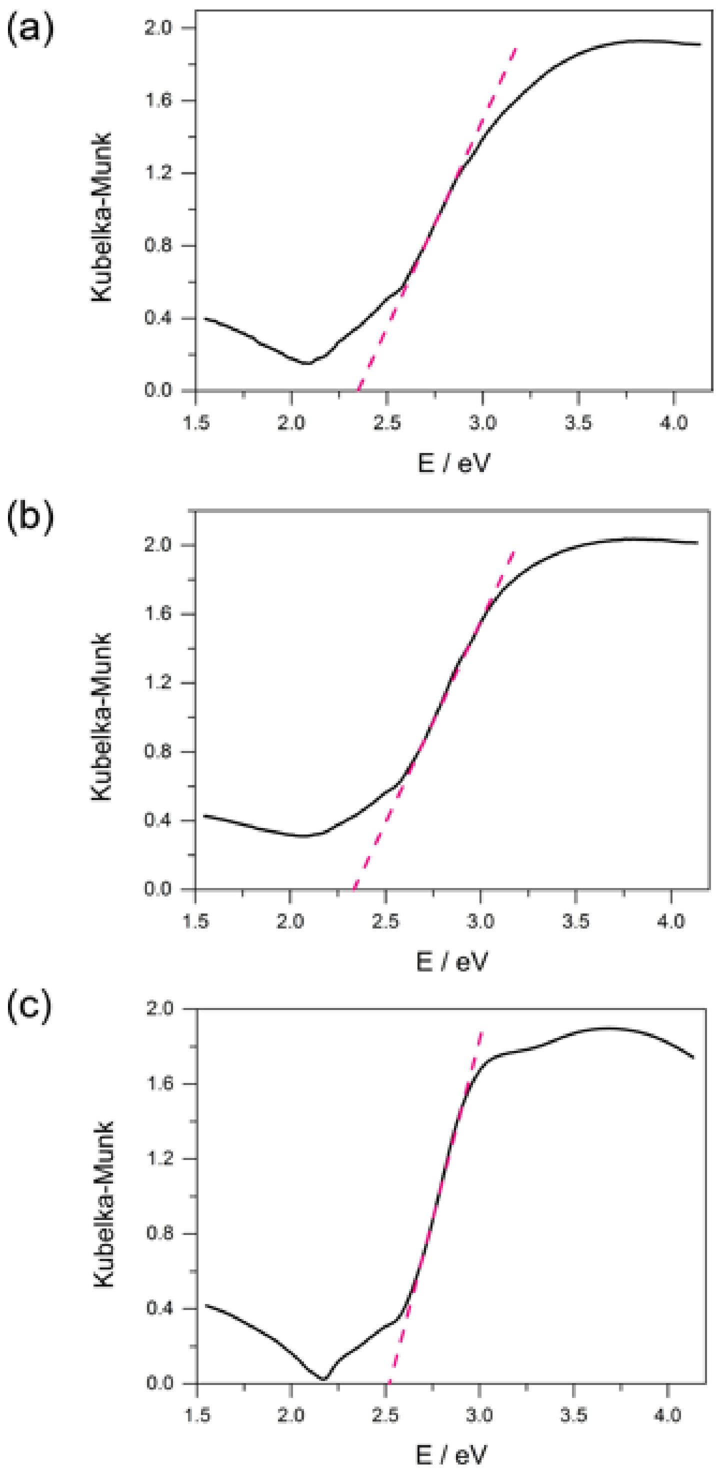

Supplementary Figure 20: Adsorption spectra of **SD/Ag44 (a), SD/Ag50 (b),** and (^{i}PrSAg)$_n$ **(c)** derived from the diffuse reflectance spectra through Kubelka-Munk function.

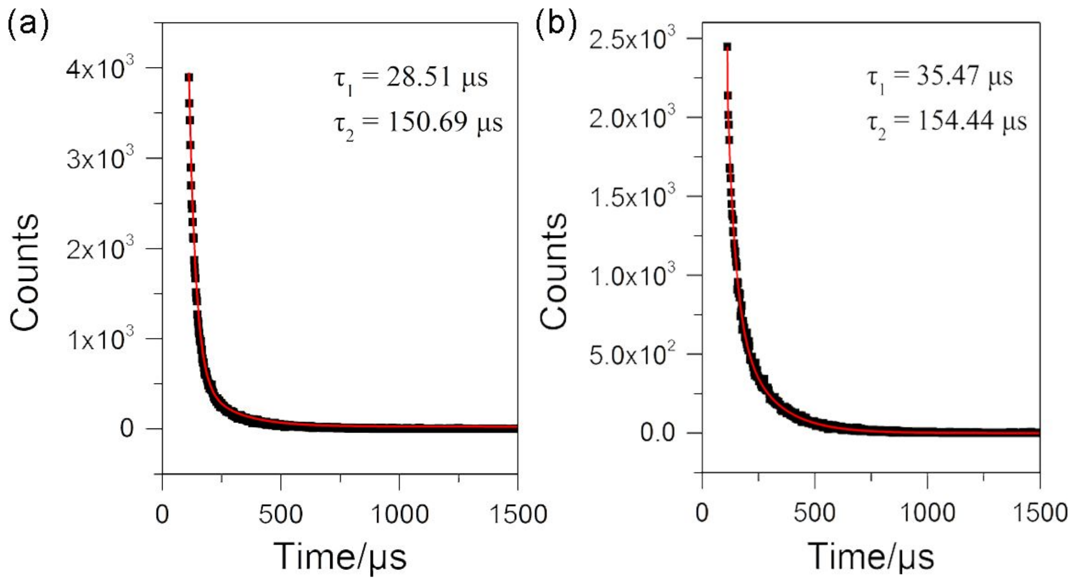

Supplementary Figure 21: Luminescent lifetimes of **SD/Ag44 (a)** and **SD/Ag50 (b)** recorded at 93 K (red lines are fitting curves).

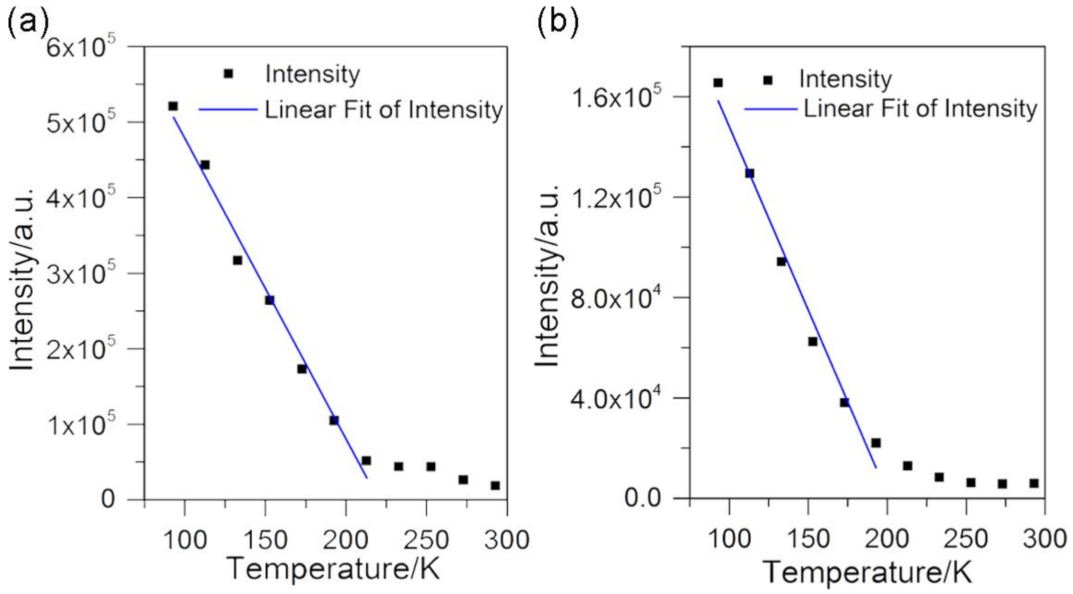

Supplementary Figure 22: The plots of temperature *vs.* maximum emission intensity of **SD/Ag44 (a)** (blue line is the linear fitting in the range of 93-213 K) and **SD/Ag50 (b)** (blue line is the linear fitting in the range of 93-193 K).

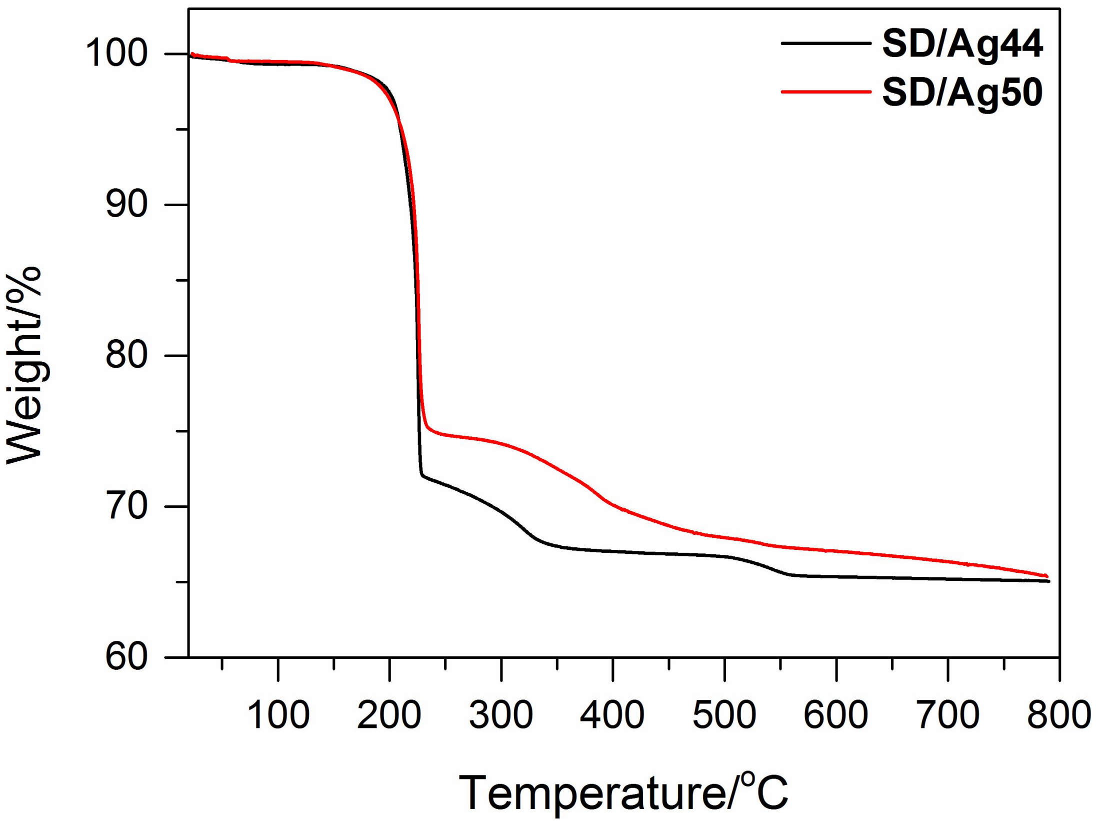

Supplementary Figure 23: The thermogravimetric analysis of **SD/Ag44** and **SD/Ag50**.

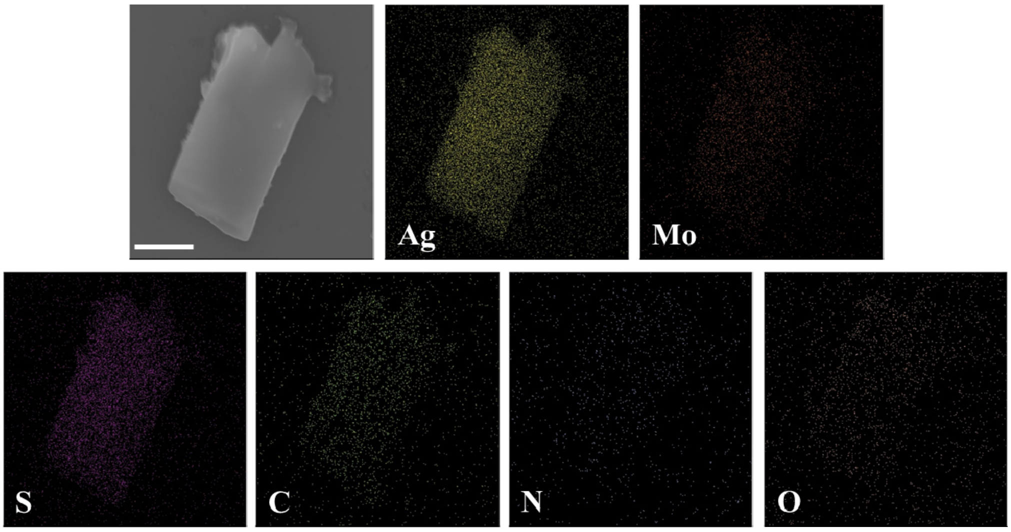

Supplementary Figure 24: SEM and elemental mapping images of **SD/Ag44**. The scale bar is 2 μm.

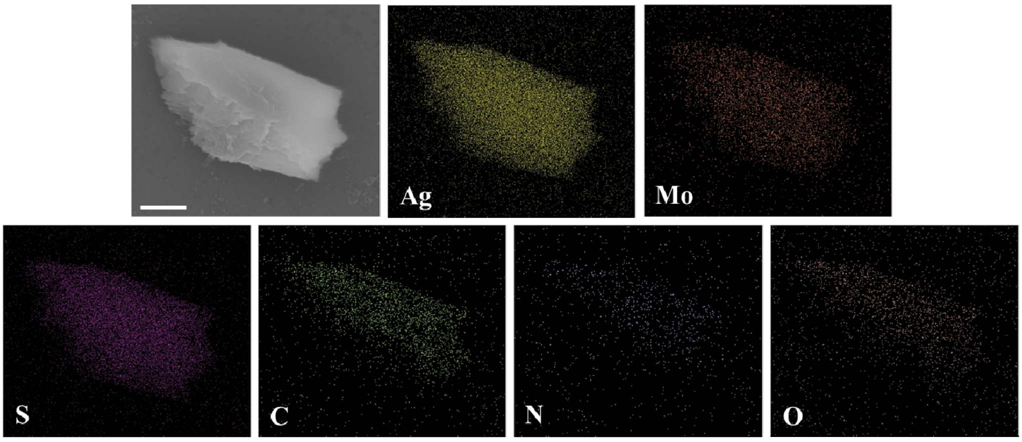

Supplementary Figure 25: SEM and elemental mapping images of **SD/Ag50**. The scale bar is 2 μm.

Supplementary Table 1: The crystal data and structure refinements for **SD/Ag44, SD/Ag50, SD/Ag50a-SD/Ag50c**.

Compound	**SD/Ag44**	**SD/Ag50**	**SD/Ag50a**	**SD/Ag50b**	**SD/Ag50c**
Empirical formula	$C_{180}H_{232}Ag_{44}Mo_6N_4O_{54}S_{20}$	$C_{210}H_{276}Ag_{50}Mo_8N_6O_{64}S_{24}$	$C_{220}H_{298}Ag_{50}Mo_8N_4O_{64}S_{24}$	$C_{214.5}H_{291}Ag_{50}Mo_8O_{64}S_{24}$	$C_{234}H_{317}Ag_{50}Mo_8N_5O_{66}S_{24}$
Formula weight	9278.80	10838.81	10953.07	10823.92	11186.37
Temperature/K	100(2)	123(2)	100.00(10)	99.99(10)	99.99(10)
Crystal system	triclinic	triclinic	orthorhombic	monoclinic	triclinic
Space group	P-1	P-1	Fddd	$P2_1/c$	P-1
a/Å	18.605(2)	19.989(3)	22.8858(7)	30.3608(4)	19.92244(16)
b/Å	20.345(2)	23.446(3)	41.4014(11)	24.5622(3)	24.62226(18)
c/Å	36.711(4)	32.734(4)	70.386(2)	40.0148(6)	34.2469(2)
α/°	84.1880(10)	98.556(2)	90	90	95.7638(6)
β/°	88.6840(10)	97.053(2)	90	96.4265(12)	94.8185(6)
γ/°	64.0240(10)	97.435(2)	90	90	98.1937(6)
Volume/ $Å^3$	12424(3)	14885(3)	66691(3)	29652.6(7)	16461.1(2)
Z	2	2	8	4	2
$\rho_{calc}g/cm^3$	2.480	2.418	2.182	2.425	2.257
μ/mm^{-1}	3.898	3.756	3.354	3.770	3.401
F(000)	8824.0	10320.0	41824.0	20640.0	10708.0
Radiation	MoKα (λ = 0.71073)	MoKα (λ = 0.71073)	MoKα (λ = 0.71073)	MoKα (λ = 0.71073)	MoKα (λ = 0.71073)
Reflections collected	185305	175911	87131	176145	193826
Independent reflections	43703 [R_{int} = 0.0419, R_{sigma} = 0.0348]	52248 [R_{int} = 0.0535, R_{sigma} = 0.0616]	16940 [R_{int} = 0.0471, R_{sigma} = 0.0389]	60245 [R_{int} = 0.0390, R_{sigma} = 0.0494]	66474 [R_{int} = 0.0334, R_{sigma} = 0.0401]
Data/parameters	43703/2817	52248/3283	16940/714	60245/3037	66474/3634
Goodness-of-fit on F^2	1.150	1.103	1.031	1.036	1.038
Final R indexes [I>=2σ (I)]	R_1 = 0.0355, wR_2 = 0.0752	R_1 = 0.0678, wR_2 = 0.1320	R_1 = 0.0889, wR_2 = 0.2223	R_1 = 0.0639, wR_2= 0.1601	R_1= 0.0407, wR_2= 0.0906
Final R indexes [all data]	R_1 = 0.0408, wR_2 = 0.0769	R_1 = 0.1198, wR_2 = 0.1723	R_1 = 0.1226, wR_2 = 0.2474	R_1 = 0.0933, wR_2 = 0.1800	R_1 = 0.0558, wR_2 = 0.0972
Largest diff. peak/hole / e $Å^{-3}$	3.48/-1.40	2.50/-3.42	4.80/-1.92	4.80/-3.47	4.75/-3.87

Supplementary Table 2: The summary of the structures and coordination modes of $Mo_6O_{22}^{8-}$ found in Ag clusters.

$Mo_6O_{22}^{8-}$	Numbers of Ag ligated by POM	Reference
$Mo_6O_{22}@Ag_{58}S_2(SC_6H_4{}^tBu)_{36}(CF_3COO)_{10}(H_2O)_8$ 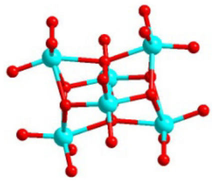	24	[8]
$Mo_6O_{22}@Ag_{40}(C{\equiv}C^tBu)_{20}(CF_3COO)_{12}$ 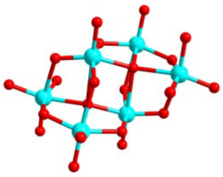	24	[9]
$[(Mo_6O_{22})_2@Ag_{60}(C{\equiv}C^tBu)_{38}](CF_3SO_3)_6$ 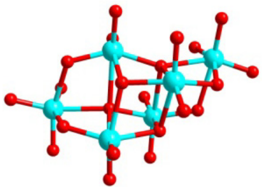	22	[10]
$[Mo_6O_{22}@Ag_{46}({}^tBuC_6H_4S)_{32}(dppm)_4(CH_3CN)_8](CF_3SO_3)_6$ 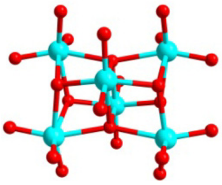	28	[11]
$[Mo_6O_{22}@Ag_{44}({}^iPrS)_{20}(PhCOO)_{16}(CH_3CN)_2]\cdot 2CH_3CN$ 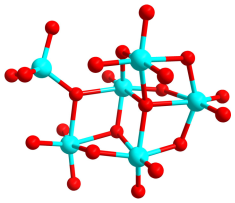	30	This work

Supplementary Table 3: The summary of the structures and coordination modes of $Mo_6O_{22}^{8-}$ found in organic-inorganic hybrid compounds.

$Mo_6O_{22}^{8-}$	Reference
$[\{Cu(2,2'\text{-}bpy)\}_6(Mo_6O_{22})][GeMo_{12}O_{40}]\cdot H_2O$ 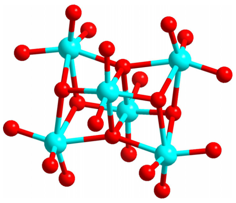	[12]
$[\{Cu(2,2'\text{-}bpy)\}_6(Mo_6O_{22})][PMo_{12}O_{40}]\cdot H_2O$ 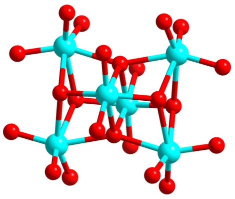	[13]
$K_2[\{Cu(phen)\}_4Mo_6O_{22}][Mo_8O_{26}]\cdot 2H_3O$ 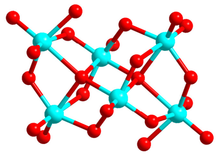	[14]
$[Cu_6(2,2'\text{-}bpy)_6(Mo_6O_{22})(SiW_{12}O_{40})]_n$ 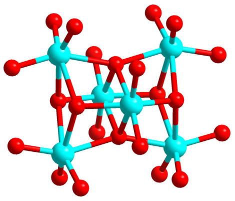	[15]

Supplementary Table 4: The formulae of the key species detected in ESI-MS of **SD/Ag44** dissolved in CH_2Cl_2.

Peak	Species	Exp. *m/z*	Sim. *m/z*
**1a**	$[\Delta({}^{i}PrS)_{32}(PhCOO)_2(CH_2Cl_2)_2(H_2O)]^{2+}$	4254.52	4254.48
**1b**	$[\Delta({}^{i}PrS)_{34}(CH_2Cl_2)_3(H_2O)_4]^{2+}$	4278.02	4277.97
**1c**	$[\Delta({}^{i}PrS)_{33}(PhCOO)(CH_2Cl_2)_3(H_2O)_4]^{2+}$	4301.02	4300.97
**1d**	$[\Delta({}^{i}PrS)_{27}(PhCOO)_7(CH_2Cl_2)_2(H_2O)]^{2+}$	4369.48	4369.49
**1e**	$[\Delta({}^{i}PrS)_{26}(PhCOO)_8(CH_2Cl_2)_2(H_2O)]^{2+}$	4392.48	4392.49
**1f**	$[\Delta({}^{i}PrS)_{22}(PhCOO)_{12}(H_2O)_3]^{2+}$	4416.98	4417.06
**1g**	$[\Delta({}^{i}PrS)_{22}(PhCOO)_{12}(CH_2Cl_2)_2(H_2O)]^{2+}$	4483.94	4484.00
**1h**	$[\Delta({}^{i}PrS)_{21}(PhCOO)_{13}(CH_2Cl_2)_2(H_2O)]^{2+}$	4506.95	4507.00
**1i**	$[\Delta({}^{i}PrS)_{23}(PhCOO)_{11}(CH_2Cl_2)_3(H_2O)_4]^{2+}$	4530.44	4530.49
**1j**	$[\Delta({}^{i}PrS)_{24}(PhCOO)_{10}(CH_2Cl_2)_6]^{2+}$	4598.91	4598.89
**1k**	$[\Delta({}^{i}PrS)_{23}(PhCOO)_{11}(CH_2Cl_2)_6]^{2+}$	4621.91	4621.89
$\Delta = Mo_6O_{22}@Ag_{44}$			

Supplementary Table 5: The formulae of the key species detected in ESI-MS of **SD/Ag50** dissolved in CH_2Cl_2.

Peak	Species	Exp. *m/z*	Sim. *m/z*
**2a**	$[\Delta(^{i}PrS)_{25}(PhCOO)_9(CH_2Cl_2)(H_2O)_3]^{2+}$	4391.01	4391.03
**2b**	$[Mo_8O_{28}@Ag_{46}(^{i}PrS)_{22}(PhCOO)_{14}(CH_2Cl_2)(H_2O)_3]^{2+}$	4832.79	4832.86
**2c**	$[Mo_8O_{28}@Ag_{47}(^{i}PrS)_{22}(PhCOO)_{15}(CH_2Cl_2)(H_2O)_3]^{2+}$	4947.25	4947.32
**2d**	$[Mo_8O_{28}@Ag_{49}(^{i}PrS)_{24}(PhCOO)_{15}]^{2+}$	5060.72	5060.76
**2e**	$[Mo_8O_{28}@Ag_{50}(^{i}PrS)_{24}(PhCOO)_{16}]^{2+}$	5175.19	5175.23
$\Delta = Mo_6O_{22}@Ag_{44}$			

Supplementary Table 6: The formulae of the key species found during the transformation from **Ag_{44}** to **Ag_{50}** clusters in CH_2Cl_2 revealed by time-dependent ESI-MS.

Peak	Species	Exp. *m/z*	Sim. *m/z*
**1l**	$[Mo_5O_{18}@Ag_{38}(^iPrS)_{18}(PhCOO)_{12}]^{2+}$	3836.31	3836.33
**1m**	$[Mo_5O_{18}@Ag_{39}(^iPrS)_{18}(PhCOO)_{13}]^{2+}$	3950.77	3950.80
**1n**	$[Mo_5O_{18}@Ag_{40}(^iPrS)_{19}(PhCOO)_{13}]^{2+}$	4042.24	4042.26
**1o**	$[Mo_5O_{18}@Ag_{40}(^iPrS)_{18}(PhCOO)_{14}]^{2+}$	4065.23	4065.26
**1p**	$[Mo_8O_{28}@Ag_{45}(^iPrS)_{22}(PhCOO)_{13}(CH_2Cl_2)]^{2+}$	4691.38	4691.37
**1q**	$[Mo_8O_{28}@Ag_{46}(^iPrS)_{22}(PhCOO)_{14}(CH_2Cl_2)]^{2+}$	4805.85	4805.84
**1r**	$[Mo_8O_{28}@Ag_{47}(^iPrS)_{22}(PhCOO)_{15}(CH_2Cl_2)]^{2+}$	4920.30	4920.31
**2d**	$[Mo_8O_{28}@Ag_{49}(^iPrS)_{24}(PhCOO)_{15}]^{2+}$	5060.71	5060.76
**2e**	$[Mo_8O_{28}@Ag_{50}(^iPrS)_{24}(PhCOO)_{16}]^{2+}$	5175.17	5175.23

Supplementary Table 7: Selected bond lengths (Å) and angles (°) for **SD/Ag44, SD/Ag50, SD/Ag50a-SD/Ag50c**.

**SD/Ag44**			
Ag1—Ag2	2.9314 (8)	Ag21—S15	2.5920 (17)
Ag1—Ag27	2.9368 (8)	Ag21—S18	2.5384 (18)
Ag1—O43	2.137 (5)	Ag22—Ag31	2.9528 (7)
Ag1—S1	2.3880 (17)	Ag22—O2	2.575 (4)
Ag2—Ag3	3.1568 (8)	Ag22—O29	2.428 (5)
Ag2—Ag14	3.0575 (8)	Ag22—S17	2.5166 (17)
Ag2—Ag27	3.2453 (7)	Ag22—S18	2.4619 (17)
Ag2—O50	2.289 (5)	Ag23—Ag31	3.0228 (7)
Ag2—S1	2.5285 (17)	Ag23—Ag32	3.0353 (7)
Ag2—S4	2.4926 (17)	Ag23—O12	2.388 (4)
Ag3—Ag7	3.1798 (8)	Ag23—S12	2.4889 (17)
Ag3—O8	2.429 (4)	Ag23—S17	2.4538 (17)
Ag3—S1	2.4285 (17)	Ag24—Ag39	2.9679 (7)
Ag3—S2	2.4467 (17)	Ag24—O15	2.451 (4)
Ag4—Ag5	2.9354 (8)	Ag24—O54	2.214 (5)
Ag4—Ag44	3.0304 (8)	Ag24—S11	2.5224 (16)
Ag4—O51	2.194 (5)	Ag25—Ag37	2.8829 (7)
Ag4—S1	2.7589 (18)	Ag25—O23	2.289 (5)
Ag4—S8	2.4513 (17)	Ag25—S5	2.4822 (16)
Ag5—Ag6	2.9854 (8)	Ag25—S11	2.4799 (16)
Ag5—Ag44	3.0479 (8)	Ag26—Ag27	3.3747 (7)
Ag5—O8	2.355 (4)	Ag26—Ag35	3.3791 (8)
Ag5—O52	2.297 (4)	Ag26—Ag36	3.3416 (8)
Ag5—S14	2.4646 (17)	Ag26—O18	2.454 (4)
Ag6—Ag43	3.0344 (8)	Ag26—S4	2.4725 (16)
Ag6—O1	2.527 (5)	Ag26—S5	2.4653 (16)
Ag6—O5	2.507 (4)	Ag27—O21	2.409 (4)
Ag6—S14	2.5392 (17)	Ag27—O42	2.582 (5)
Ag6—S15	2.4703 (18)	Ag27—O44	2.210 (4)
Ag7—Ag8	2.9061 (7)	Ag27—S4	2.4751 (16)
Ag7—Ag9	3.2847 (8)	Ag28—Ag29	2.9320 (8)
Ag7—O7	2.393 (4)	Ag28—Ag35	3.0818 (8)
Ag7—O35	2.290 (5)	Ag28—Ag44	2.9943 (8)
Ag7—O52	2.524 (5)	Ag28—O20	2.417 (4)
Ag7—S2	2.4887 (18)	Ag28—S8	2.4481 (17)
Ag8—Ag12	3.1335 (8)	Ag28—S9	2.5087 (16)
Ag8—Ag13	3.1705 (8)	Ag29—Ag34	3.3119 (8)

Ag8—O36	2.527 (5)	Ag29—O22	2.521 (4)
Ag8—O37	2.295 (5)	Ag29—O46	2.114 (5)
Ag8—S2	2.5640 (18)	Ag29—S9	2.4397 (17)
Ag8—S7	2.5803 (17)	Ag30—Ag33	3.2026 (8)
Ag9—Ag10	2.9503 (7)	Ag30—Ag42	3.0524 (9)
Ag9—Ag11	3.0241 (8)	Ag30—O13	2.255 (4)
Ag9—O7	2.491 (4)	Ag30—O48	2.455 (5)
Ag9—O35	2.544 (4)	Ag30—S19	2.4279 (17)
Ag9—S7	2.4744 (16)	Ag31—Ag32	2.8272 (7)
Ag9—S15	2.4536 (16)	Ag31—Ag42	3.2676 (8)
Ag10—Ag11	2.9877 (8)	Ag31—O28	2.294 (5)
Ag10—Ag20	2.8359 (8)	Ag31—O29	2.432 (5)
Ag10—Ag21	3.1511 (8)	Ag31—S17	2.5750 (17)
Ag10—O34	2.284 (5)	Ag31—S20	2.4857 (17)
Ag10—S15	2.4733 (18)	Ag32—Ag33	2.9876 (7)
Ag10—S16	2.6812 (18)	Ag32—Ag40	3.0510 (8)
Ag11—Ag12	3.1388 (8)	Ag32—O27	2.355 (5)
Ag11—Ag18	3.1695 (7)	Ag32—S12	2.4833 (16)
Ag11—O6	2.424 (4)	Ag32—S20	2.5750 (17)
Ag11—S7	2.4900 (16)	Ag33—Ag40	2.9909 (7)
Ag11—S16	2.4501 (17)	Ag33—O13	2.545 (4)
Ag12—Ag16	3.0022 (7)	Ag33—O48	2.575 (5)
Ag12—O9	2.556 (4)	Ag33—S13	2.4421 (17)
Ag12—O38	2.374 (4)	Ag33—S20	2.4263 (17)
Ag12—S6	2.5241 (17)	Ag34—Ag38	3.2993 (8)
Ag12—S7	2.4914 (16)	Ag34—O13	2.484 (4)
Ag13—Ag14	3.0995 (8)	Ag34—O47	2.538 (5)
Ag13—O9	2.476 (4)	Ag34—S9	2.4860 (17)
Ag13—O19	2.600 (4)	Ag34—S13	2.5232 (17)
Ag13—S2	2.4472 (17)	Ag35—Ag36	3.2629 (8)
Ag13—S3	2.4214 (17)	Ag35—O18	2.279 (4)
Ag14—Ag15	3.3464 (8)	Ag35—O42	2.319 (5)
Ag14—O49	2.285 (5)	Ag35—S9	2.4334 (16)
Ag14—S3	2.5369 (17)	Ag36—O16	2.449 (4)
Ag14—S4	2.5748 (16)	Ag36—O41	2.272 (5)
Ag15—Ag16	3.3204 (8)	Ag36—S5	2.5899 (16)
Ag15—Ag26	2.9829 (7)	Ag36—S10	2.4626 (17)
Ag15—O17	2.536 (4)	Ag37—Ag39	3.3307 (8)
Ag15—S3	2.4231 (16)	Ag37—O24	2.200 (4)
Ag15—S5	2.4335 (16)	Ag37—O25	2.511 (4)
Ag16—Ag17	2.9971 (7)	Ag37—S10	2.4113 (16)
Ag16—O38	2.576 (4)	Ag38—Ag39	3.2869 (7)
Ag16—O39	2.346 (4)	Ag38—Ag40	3.1178 (7)

Ag16—S3	2.5494 (17)	Ag38—O14	2.483 (4)
Ag16—S6	2.5441 (16)	Ag38—S10	2.3641 (17)
Ag17—Ag24	2.9317 (7)	Ag38—S13	2.4073 (16)
Ag17—Ag25	3.0783 (8)	Ag39—Ag40	3.0505 (8)
Ag17—O17	2.496 (4)	Ag39—O14	2.420 (4)
Ag17—O40	2.391 (4)	Ag39—O25	2.300 (4)
Ag17—S6	2.5381 (17)	Ag39—S11	2.6271 (16)
Ag17—S11	2.5868 (16)	Ag39—S12	2.5865 (17)
Ag18—Ag24	2.9570 (7)	Ag40—N1	2.395 (6)
Ag18—O4	2.414 (4)	Ag40—O26	2.331 (5)
Ag18—O6	2.482 (4)	Ag40—S12	2.5854 (16)
Ag18—O15	2.541 (4)	Ag40—S13	2.6396 (17)
Ag18—O53	2.437 (5)	Ag41—Ag42	3.0493 (8)
Ag18—S6	2.5181 (16)	Ag41—N2	2.338 (7)
Ag19—Ag20	3.0319 (8)	Ag41—O31	2.382 (6)
Ag19—Ag22	3.0393 (8)	Ag41—S18	2.5336 (18)
Ag19—O4	2.427 (4)	Ag41—S19	2.5129 (18)
Ag19—O53	2.507 (5)	Ag42—O2	2.433 (4)
Ag19—S16	2.4698 (16)	Ag42—O30	2.293 (5)
Ag19—S17	2.4232 (16)	Ag42—S19	2.6142 (18)
Ag20—Ag21	2.9636 (8)	Ag42—S20	2.6598 (17)
Ag20—Ag22	2.9559 (8)	Ag43—S14	2.4177 (18)
Ag20—O33	2.434 (7)	Ag43—S19	2.4077 (18)
Ag20—S16	2.4701 (17)	Ag44—O45	2.349 (5)
Ag20—S18	2.4378 (17)	Ag44—S8	2.4908 (18)
Ag21—Ag41	3.1444 (8)	Ag44—S14	2.5277 (19)
Ag21—O32	2.303 (5)		
O4—Ag1—S1	163.83(13)	O29—Ag22—S17	93.86(13)
O50—Ag2—S1	93.37(14)	O29—Ag22—S18	102.92(13)
O50—Ag2—S4	117.16(14)	S17—Ag22—O2	92.28(11)
S4—Ag2—S1	149.47(5)	S18—Ag22—O2	88.34(11)
O8—Ag3—S2	102.15(10)	S18—Ag22—S17	162.45(6)
S1—Ag3—O8	114.81(10)	O12—Ag23—S12	88.57(11)
S1—Ag3—S2	141.98(6)	O12—Ag23—S17	110.86(11)
O51—Ag4—S1	94.58(14)	S17—Ag23—S12	155.25(6)
O51—Ag4—S8	160.76(13)	O15—Ag24—S11	101.21(10)
S8—Ag4—S1	101.86(6)	O54—Ag24—O15	134.99(16)
O8—Ag5—S14	134.99(10)	O54—Ag24—S11	123.77(13)
O52—Ag5—O8	92.23(15)	O23—Ag25—S5	99.40(13)
O52—Ag5—S14	132.53(13)	O23—Ag25—S11	115.78(13)
O1—Ag6—S14	114.81(12)	S11—Ag25—S5	144.32(5)
O5—Ag6—O1	75.43(14)	O18—Ag26—S4	107.44(10)
O5—Ag6—S14	77.06(10)	O18—Ag26—S5	108.61(10)

S15—Ag6—O1	86.15(12)	S5—Ag26—S4	139.47(6)
S15—Ag6—O5	132.94(10)	O21—Ag27—O42	106.43(14)
S15—Ag6—S14	148.62(6)	O21—Ag27—S4	89.63(11)
O7—Ag7—O52	80.89(14)	O44—Ag27—O21	109.17(16)
O7—Ag7—S2	112.07(11)	O44—Ag27—O42	90.96(15)
O35—Ag7—O7	96.88(15)	O44—Ag27—S4	158.24(13)
O35—Ag7—O52	112.34(16)	S4—Ag27—O42	94.14(10)
O35—Ag7—S2	135.28(12)	O20—Ag28—S8	97.52(10)
S2—Ag7—O52	105.55(13)	O20—Ag28—S9	89.31(10)
O36—Ag8—S2	104.67(12)	S8—Ag28—S9	169.60(5)
O36—Ag8—S7	81.62(11)	O46—Ag29—O22	97.01(19)
O37—Ag8—O36	111.54(16)	O46—Ag29—S9	166.10(17)
O37—Ag8—S2	109.01(14)	S9—Ag29—O22	96.87(10)
O37—Ag8—S7	109.31(14)	O13—Ag30—O48	97.04(16)
S2—Ag8—S7	135.29(5)	O13—Ag30—S19	145.44(11)
O7—Ag9—O35	88.20(14)	S19—Ag30—O48	114.35(13)
S7—Ag9—O7	94.02(10)	O28—Ag31—O29	92.25(19)
S7—Ag9—O35	92.53(11)	O28—Ag31—S17	94.07(15)
S15—Ag9—Ag7	120.57(5)	O28—Ag31—S20	111.40(14)
S15—Ag9—O7	92.97(11)	O29—Ag31—S17	92.33(13)
S15—Ag9—O35	115.09(11)	O29—Ag31—S20	106.11(14)
S15—Ag9—S7	151.70(6)	S20—Ag31—S17	147.40(6)
O34—Ag10—S15	121.74(15)	O27—Ag32—S12	119.08(13)
O34—Ag10—S16	99.08(15)	O27—Ag32—S20	101.71(13)
S15—Ag10—S16	138.00(6)	S12—Ag32—S20	137.30(5)
O6—Ag11—S7	91.97(10)	O13—Ag33—O48	87.22(16)
O6—Ag11—S16	126.15(10)	S13—Ag33—O13	81.16(10)
S16—Ag11—S7	139.67(6)	S13—Ag33—O48	98.21(13)
O38—Ag12—O9	113.15(15)	S20—Ag33—O13	101.33(10)
O38—Ag12—S6	95.47(12)	S20—Ag33—O48	87.42(13)
O38—Ag12—S7	113.32(12)	S20—Ag33—S13	173.98(6)
S6—Ag12—O9	101.21(10)	O13—Ag34—O47	91.28(18)
S7—Ag12—O9	84.45(10)	O13—Ag34—S9	129.49(10)
S7—Ag12—S6	145.92(6)	O13—Ag34—S13	80.80(10)
O9—Ag13—O19	77.61(13)	S9—Ag34—O47	90.67(14)
S2—Ag13—O9	82.81(10)	S9—Ag34—S13	147.80(6)
S2—Ag13—O19	97.65(10)	S13—Ag34—O47	100.37(13)
S3—Ag13—O9	119.56(10)	O18—Ag35—O42	97.18(15)
S3—Ag13—O19	86.37(10)	O18—Ag35—S9	132.87(11)
S3—Ag13—S2	157.56(6)	O42—Ag35—S9	120.16(11)
O49—Ag14—S3	109.26(13)	O16—Ag36—S5	80.07(11)
O49—Ag14—S4	109.09(13)	O16—Ag36—S10	93.88(11)
S3—Ag14—S4	140.47(5)	O41—Ag36—O16	133.13(18)

S3—Ag15—O17	92.14(11)	O41—Ag36—S5	110.50(15)
S3—Ag15—S5	163.08(6)	O41—Ag36—S10	112.12(13)
S5—Ag15—O17	94.26(10)	S10—Ag36—S5	125.76(5)
O39—Ag16—O38	102.56(15)	O24—Ag37—O25	110.16(16)
O39—Ag16—S3	90.66(12)	O24—Ag37—S10	146.94(13)
O39—Ag16—S6	113.33(12)	S10—Ag37—O25	94.16(11)
S3—Ag16—O38	104.47(11)	S10—Ag38—O14	109.58(11)
S6—Ag16—O38	90.18(11)	S10—Ag38—S13	163.10(6)
S6—Ag16—S3	148.68(5)	S13—Ag38—O14	87.31(11)
O17—Ag17—S6	83.23(11)	O14—Ag39—S11	97.06(11)
O17—Ag17—S11	110.85(10)	O14—Ag39—S12	91.75(11)
O40—Ag17—O17	94.66(14)	O25—Ag39—O14	132.93(15)
O40—Ag17—S6	120.96(11)	O25—Ag39—S11	98.27(11)
O40—Ag17—S11	102.55(11)	O25—Ag39—S12	108.27(12)
S6—Ag17—S11	133.36(5)	S12—Ag39—S11	133.82(5)
O4—Ag18—O6	69.43(14)	N1—Ag40—S12	109.09(17)
O4—Ag18—O15	109.74(14)	N1—Ag40—S13	103.28(17)
O4—Ag18—O53	78.92(15)	O26—Ag40—N1	89.6(2)
O4—Ag18—S6	156.93(11)	O26—Ag40—S12	109.24(13)
O6—Ag18—O15	93.25(13)	O26—Ag40—S13	101.64(13)
O6—Ag18—S6	103.04(10)	S12—Ag40—S13	134.72(5)
O53—Ag18—O6	136.35(15)	N2—Ag41—O31	98.9(3)
O53—Ag18—O15	126.12(14)	N2—Ag41—S18	101.52(19)
O53—Ag18—S6	94.38(11)	N2—Ag41—S19	113.77(18)
S6—Ag18—O15	92.12(10)	O31—Ag41—S18	113.85(16)
O4—Ag19—O53	77.34(15)	O31—Ag41—S19	93.61(15)
O4—Ag19—S16	77.01(11)	S19—Ag41—S18	131.08(6)
S16—Ag19—O53	98.58(11)	O2—Ag42—S19	87.03(11)
S17—Ag19—O4	104.04(11)	O2—Ag42—S20	92.41(11)
S17—Ag19—O53	98.94(11)	O30—Ag42—O2	127.78(18)
S17—Ag19—S16	162.23(6)	O30—Ag42—S19	121.84(14)
O33—Ag20—S16	96.49(18)	O30—Ag42—S20	100.39(14)
O33—Ag20—S18	105.60(19)	S19—Ag42—S20	126.02(6)
S18—Ag20—S16	156.86(6)	S19—Ag43—S14	173.58(6)
O32—Ag21—S15	91.68(13)	O45—Ag44—S8	114.82(13)
O32—Ag21—S18	116.44(13)	O45—Ag44—S14	88.80(13)
S18—Ag21—S15	151.83(6)	S8—Ag44—S14	151.10(6)
O29—Ag22—O2	105.01(16)		
**SD/Ag50**			
Ag1—Ag9	3.139 (4)	Ag24—Ag25	3.166 (2)
Ag1—Ag23	2.929 (4)	Ag24—S15	2.381 (4)
Ag1—O21	2.480 (11)	Ag24—S17	2.391 (4)
Ag1—O30	2.29 (3)	Ag25—Ag27	3.294 (2)

Ag1—S17	2.654 (6)	Ag25—O23	2.426 (11)
Ag1—S18	2.688 (6)	Ag25—O64	2.499 (12)
Ag2—Ag45	3.318 (4)	Ag25—S13	2.518 (4)
Ag2—Ag46	3.126 (4)	Ag25—S15	2.554 (5)
Ag2—O10	2.516 (11)	Ag26—Ag32	2.867 (2)
Ag2—O46	2.518 (11)	Ag26—O24	2.363 (10)
Ag2—S19	2.248 (7)	Ag26—O64	2.310 (10)
Ag2—S20	2.527 (5)	Ag26—S1	2.775 (5)
Ag3—Ag4	3.049 (2)	Ag26—S24	2.549 (4)
Ag3—Ag5	2.992 (2)	Ag27—Ag28	2.9930 (18)
Ag3—N2	2.39 (3)	Ag27—Ag29	2.8620 (19)
Ag3—S10	2.501 (4)	Ag27—O62	2.303 (12)
Ag3—S14	2.492 (5)	Ag27—S1	2.751 (5)
Ag4—Ag5	3.0857 (19)	Ag27—S13	2.521 (5)
Ag4—Ag17	3.1063 (19)	Ag28—Ag29	3.371 (2)
Ag4—Ag21	3.301 (2)	Ag28—O54	2.353 (11)
Ag4—O39	2.319 (13)	Ag28—S2	2.503 (4)
Ag4—S12	2.549 (5)	Ag28—S13	2.467 (4)
Ag4—S14	2.570 (5)	Ag29—Ag30	3.0266 (17)
Ag5—Ag7	3.3238 (19)	Ag29—Ag31	2.8996 (19)
Ag5—O37	2.301 (14)	Ag29—O61	2.279 (11)
Ag5—S10	2.559 (5)	Ag29—S1	2.584 (4)
Ag5—S12	2.490 (4)	Ag29—S2	2.455 (5)
Ag6—Ag7	3.123 (2)	Ag30—Ag31	3.1603 (18)
Ag6—Ag18	2.9967 (17)	Ag30—O3	2.417 (9)
Ag6—O27	2.414 (10)	Ag30—O56	2.513 (11)
Ag6—O53	2.470 (11)	Ag30—S2	2.469 (4)
Ag6—S9	2.471 (5)	Ag30—S3	2.482 (5)
Ag6—S10	2.503 (5)	Ag31—Ag32	2.9036 (18)
Ag7—Ag16	2.9325 (19)	Ag31—Ag35	3.3714 (19)
Ag7—Ag18	2.957 (2)	Ag31—O2	2.569 (9)
Ag7—O28	2.531 (9)	Ag31—O59	2.497 (13)
Ag7—O34	2.463 (15)	Ag31—S1	2.517 (4)
Ag7—S8	2.530 (4)	Ag31—S3	2.468 (4)
Ag7—S10	2.489 (4)	Ag32—Ag33	3.0117 (17)
Ag8—Ag15	3.3365 (19)	Ag32—Ag34	3.2246 (19)
Ag8—Ag17	3.1248 (19)	Ag32—O2	2.520 (9)
Ag8—O28	2.479 (11)	Ag32—O20	2.480 (11)
Ag8—S11	2.462 (4)	Ag32—O60	2.274 (13)
Ag8—S12	2.490 (4)	Ag32—S1	2.608 (4)
Ag9—Ag14	3.1089 (18)	Ag33—Ag34	3.1715 (18)
Ag9—Ag23	3.3246 (19)	Ag33—S21	2.401 (4)
Ag9—O21	2.394 (13)	Ag33—S24	2.426 (4)

Ag9—O29	2.46 (3)	Ag34—Ag37	3.129 (2)
Ag9—S11	2.657 (5)	Ag34—O2	2.558 (9)
Ag9—S16	2.532 (4)	Ag34—S21	2.501 (4)
Ag10—Ag11	3.0031 (17)	Ag34—S23	2.525 (4)
Ag10—Ag24	3.243 (2)	Ag35—Ag36	2.9664 (19)
Ag10—Ag26	2.893 (2)	Ag35—Ag50	2.996 (2)
Ag10—O23	2.585 (10)	Ag35—O58	2.357 (14)
Ag10—O63	2.401 (12)	Ag35—S3	2.544 (4)
Ag10—S17	2.484 (5)	Ag35—S23	2.487 (4)
Ag10—S24	2.531 (5)	Ag36—Ag37	3.211 (2)
Ag11—Ag12	2.9286 (16)	Ag36—Ag38	3.116 (2)
Ag11—Ag33	2.9089 (18)	Ag36—Ag50	3.067 (2)
Ag11—O43	2.289 (10)	Ag36—O52	2.461 (13)
Ag11—S18	2.502 (5)	Ag36—O57	2.278 (13)
Ag11—S24	2.545 (4)	Ag36—S4	2.562 (4)
Ag12—Ag13	3.330 (2)	Ag36—S23	2.644 (4)
Ag12—Ag33	2.9180 (17)	Ag37—Ag38	3.275 (2)
Ag12—O44	2.428 (11)	Ag37—Ag43	3.133 (2)
Ag12—O45	2.446 (10)	Ag37—S22	2.469 (4)
Ag12—S18	2.576 (5)	Ag37—S23	2.470 (5)
Ag12—S21	2.573 (4)	Ag38—Ag42	3.119 (2)
Ag13—Ag14	3.285 (2)	Ag38—Ag43	3.191 (2)
Ag13—O17	2.503 (10)	Ag38—O1	2.495 (10)
Ag13—O19	2.498 (10)	Ag38—S4	2.518 (5)
Ag13—S18	2.458 (5)	Ag38—S5	2.494 (6)
Ag13—S19	2.484 (5)	Ag39—Ag40	2.9623 (18)
Ag14—Ag15	3.0107 (19)	Ag39—O3	2.412 (9)
Ag14—Ag46	2.9287 (18)	Ag39—O56	2.392 (10)
Ag14—O17	2.535 (11)	Ag39—S4	2.560 (4)
Ag14—S11	2.425 (5)	Ag39—S6	2.543 (4)
Ag14—S19	2.435 (5)	Ag40—Ag41	3.1007 (19)
Ag15—Ag16	3.249 (2)	Ag40—O55	2.345 (11)
Ag15—Ag46	2.912 (2)	Ag40—S6	2.498 (4)
Ag15—O31	2.36 (2)	Ag40—S9	2.528 (4)
Ag15—S7	2.566 (4)	Ag41—Ag47	3.157 (2)
Ag15—S11	2.495 (4)	Ag41—O35	2.332 (14)
Ag16—Ag47	2.8531 (19)	Ag41—S6	2.477 (4)
Ag16—O15	2.370 (11)	Ag41—S8	2.555 (4)
Ag16—O33	2.290 (14)	Ag42—O8	2.403 (9)
Ag16—S7	2.917 (4)	Ag42—S5	2.556 (4)
Ag16—S8	2.573 (5)	Ag42—S6	2.477 (4)
Ag17—Ag21	3.278 (2)	Ag43—Ag44	3.216 (2)
Ag17—Ag22	3.3087 (19)	Ag43—Ag49	2.8775 (19)

Ag17—S12	2.533 (5)	Ag43—O51	2.238 (13)
Ag17—S16	2.513 (4)	Ag43—S5	2.511 (5)
Ag18—Ag40	3.0134 (19)	Ag43—S22	2.633 (4)
Ag18—Ag41	2.8622 (17)	Ag44—Ag49	2.8769 (18)
Ag18—O36	2.340 (14)	Ag44—O48	2.386 (11)
Ag18—S8	2.537 (5)	Ag44—O49	2.487 (16)
Ag18—S9	2.502 (5)	Ag44—S5	2.469 (6)
Ag19—Ag28	3.3594 (18)	Ag44—S20	2.568 (5)
Ag19—Ag30	3.281 (2)	Ag45—Ag46	2.851 (2)
Ag19—Ag40	3.2481 (17)	Ag45—Ag47	2.936 (2)
Ag19—O11	2.544 (9)	Ag45—O8	2.478 (10)
Ag19—O14	2.525 (9)	Ag45—O49	2.497 (16)
Ag19—S2	2.467 (5)	Ag45—S7	2.456 (4)
Ag19—S9	2.454 (5)	Ag45—S20	2.450 (4)
Ag20—Ag21	3.325 (2)	Ag46—O32	2.271 (17)
Ag20—Ag28	3.047 (2)	Ag46—S7	2.638 (5)
Ag20—O27	2.488 (9)	Ag46—S19	2.533 (5)
Ag20—O53	2.328 (11)	Ag47—O9	2.400 (9)
Ag20—S13	2.529 (5)	Ag47—O50	2.300 (14)
Ag20—S14	2.598 (5)	Ag47—S7	2.623 (5)
Ag21—Ag25	3.356 (2)	Ag47—S8	2.876 (4)
Ag21—S14	2.459 (5)	Ag48—Ag49	3.0139 (18)
Ag21—S15	2.486 (5)	Ag48—O10	2.510 (11)
Ag22—Ag23	2.9138 (19)	Ag48—O45	2.238 (11)
Ag22—Ag24	2.945 (2)	Ag48—S21	2.766 (4)
Ag22—O40	2.421 (13)	Ag48—S22	2.455 (4)
Ag22—O41	2.289 (12)	Ag49—O47	2.492 (10)
Ag22—S15	2.656 (5)	Ag49—S20	2.429 (5)
Ag22—S16	2.616 (5)	Ag49—S22	2.433 (5)
Ag23—Ag24	3.017 (2)	Ag50—N1	2.32 (3)
Ag23—O42	2.339 (12)	Ag50—S3	2.468 (4)
Ag23—S16	2.493 (5)	Ag50—S4	2.447 (6)
Ag23—S17	2.567 (6)		
O21—Ag1—S17	85.1 (3)	O64—Ag25—S13	100.6 (3)
O21—Ag1—S18	99.4 (3)	O64—Ag25—S15	125.9 (3)
O30—Ag1—O21	124.7 (8)	S13—Ag25—S15	116.25 (15)
O30—Ag1—S17	110.7 (7)	O24—Ag26—S1	74.3 (3)
O30—Ag1—S18	116.6 (7)	O24—Ag26—S24	141.7 (3)
S17—Ag1—S18	116.7 (2)	O64—Ag26—O24	92.9 (3)
O10—Ag2—O46	105.4 (4)	O64—Ag26—S1	92.1 (3)
O10—Ag2—S20	87.3 (3)	O64—Ag26—S24	120.3 (3)
O46—Ag2—S20	110.2 (3)	S24—Ag26—S1	118.90 (15)
S19—Ag2—O10	109.9 (3)	O62—Ag27—S1	90.4 (5)

S19—Ag2—O46	96.7 (3)	O62—Ag27—S13	113.7 (5)
S19—Ag2—S20	143.1 (2)	S13—Ag27—S1	154.85 (14)
N2—Ag3—S10	116.8 (8)	O54—Ag28—S2	93.7 (3)
N2—Ag3—S14	103.2 (8)	O54—Ag28—S13	113.7 (3)
S14—Ag3—S10	132.07 (15)	S13—Ag28—S2	152.26 (15)
O39—Ag4—S12	113.8 (4)	O61—Ag29—S1	95.1 (3)
O39—Ag4—S14	105.3 (4)	O61—Ag29—S2	119.5 (3)
S12—Ag4—S14	132.76 (15)	S2—Ag29—S1	142.78 (15)
O37—Ag5—S10	101.9 (4)	O3—Ag30—O56	76.5 (3)
O37—Ag5—S12	110.9 (4)	O3—Ag30—S2	116.0 (3)
S12—Ag5—S10	143.08 (17)	O3—Ag30—S3	90.4 (3)
O27—Ag6—O53	77.2 (3)	S2—Ag30—O56	103.0 (3)
O27—Ag6—S9	114.6 (3)	S2—Ag30—S3	142.85 (15)
O27—Ag6—S10	89.2 (3)	S3—Ag30—O56	108.6 (3)
O53—Ag6—S9	106.8 (3)	O59—Ag31—O2	86.3 (3)
O53—Ag6—S10	100.4 (3)	O59—Ag31—S1	107.4 (3)
S9—Ag6—S10	146.97 (15)	S1—Ag31—O2	97.3 (2)
O34—Ag7—O28	86.2 (4)	S3—Ag31—O2	99.3 (2)
O34—Ag7—S8	101.9 (3)	S3—Ag31—O59	92.7 (3)
O34—Ag7—S10	95.4 (3)	S3—Ag31—S1	154.64 (14)
S8—Ag7—O28	104.3 (2)	O2—Ag32—S1	96.2 (2)
S10—Ag7—O28	100.2 (2)	O20—Ag32—O2	70.0 (3)
S10—Ag7—S8	150.75 (15)	O20—Ag32—S1	105.9 (2)
O28—Ag8—S12	94.6 (2)	O60—Ag32—O2	97.5 (4)
S11—Ag8—O28	131.9 (3)	O60—Ag32—O20	126.3 (4)
S11—Ag8—S12	131.17 (15)	O60—Ag32—S1	127.7 (3)
O21—Ag9—O29	118.3 (7)	S21—Ag33—S24	171.98 (16)
O21—Ag9—S11	123.8 (3)	S21—Ag34—O2	129.7 (3)
O21—Ag9—S16	99.0 (3)	S21—Ag34—S23	132.69 (14)
O29—Ag9—S11	94.9 (7)	S23—Ag34—O2	93.5 (2)
O29—Ag9—S16	114.0 (6)	O58—Ag35—S3	92.8 (3)
S16—Ag9—S11	107.30 (15)	O58—Ag35—S23	116.4 (4)
O63—Ag10—O23	96.1 (5)	S23—Ag35—S3	144.52 (16)
O63—Ag10—S17	104.9 (5)	O52—Ag36—S4	104.4 (4)
O63—Ag10—S24	98.7 (5)	O52—Ag36—S23	94.4 (3)
S17—Ag10—O23	88.9 (3)	O57—Ag36—O52	107.5 (5)
S17—Ag10—S24	148.03 (14)	O57—Ag36—S4	110.7 (4)
S24—Ag10—O23	109.9 (3)	O57—Ag36—S23	102.8 (3)
O43—Ag11—S18	109.4 (3)	S4—Ag36—S23	133.79 (14)
O43—Ag11—S24	102.6 (3)	S22—Ag37—S23	147.75 (14)
S18—Ag11—S24	144.62 (13)	O1—Ag38—S4	78.6 (3)
O44—Ag12—O45	108.8 (4)	S5—Ag38—O1	138.9 (3)
O44—Ag12—S18	91.3 (3)	S5—Ag38—S4	139.05 (15)

O44—Ag12—S21	110.3 (3)	O3—Ag39—S4	100.9 (3)
O45—Ag12—S18	107.3 (3)	O3—Ag39—S6	130.4 (3)
O45—Ag12—S21	86.2 (3)	O56—Ag39—O3	78.9 (3)
S21—Ag12—S18	149.78 (13)	O56—Ag39—S4	105.1 (3)
O19—Ag13—O17	70.3 (3)	O56—Ag39—S6	123.1 (3)
S18—Ag13—O17	120.9 (3)	S6—Ag39—S4	112.29 (15)
S18—Ag13—O19	83.3 (3)	O55—Ag40—S6	110.8 (3)
S18—Ag13—S19	146.67 (18)	O55—Ag40—S9	95.0 (3)
S19—Ag13—O17	82.5 (3)	S6—Ag40—S9	154.16 (15)
S19—Ag13—O19	129.2 (3)	O35—Ag41—S6	106.0 (5)
S11—Ag14—O17	95.7 (3)	O35—Ag41—S8	105.4 (4)
S11—Ag14—S19	166.78 (18)	S6—Ag41—S8	143.58 (13)
S19—Ag14—O17	82.8 (3)	O8—Ag42—S5	101.1 (3)
O31—Ag15—S7	95.7 (5)	O8—Ag42—S6	135.2 (2)
O31—Ag15—S11	110.2 (5)	S6—Ag42—S5	120.31 (15)
S11—Ag15—S7	148.76 (17)	O51—Ag43—S5	114.4 (4)
O15—Ag16—S7	70.4 (3)	O51—Ag43—S22	106.6 (4)
O15—Ag16—S8	127.1 (3)	S5—Ag43—S22	134.98 (14)
O33—Ag16—O15	110.0 (4)	O48—Ag44—O49	118.0 (5)
O33—Ag16—S7	94.3 (3)	O48—Ag44—S5	113.4 (4)
O33—Ag16—S8	121.9 (3)	O48—Ag44—S20	94.8 (4)
S8—Ag16—S7	112.29 (13)	O49—Ag44—S20	86.3 (4)
S16—Ag17—S12	140.44 (17)	S5—Ag44—O49	101.2 (5)
O36—Ag18—S8	110.0 (5)	S5—Ag44—S20	141.72 (15)
O36—Ag18—S9	97.8 (5)	O8—Ag45—O49	79.7 (5)
S9—Ag18—S8	148.43 (13)	S7—Ag45—O8	111.9 (3)
O14—Ag19—O11	69.8 (3)	S7—Ag45—O49	107.2 (4)
S2—Ag19—O11	133.8 (2)	S20—Ag45—O8	89.1 (3)
S2—Ag19—O14	82.5 (2)	S20—Ag45—O49	88.7 (4)
S9—Ag19—O11	82.1 (2)	S20—Ag45—S7	155.35 (18)
S9—Ag19—O14	118.4 (3)	O32—Ag46—S7	103.3 (5)
S9—Ag19—S2	144.12 (14)	O32—Ag46—S19	112.1 (5)
O27—Ag20—S13	123.5 (2)	S19—Ag46—S7	142.20 (16)
O27—Ag20—S14	102.1 (3)	O9—Ag47—S7	116.8 (2)
O53—Ag20—O27	78.5 (4)	O9—Ag47—S8	71.6 (2)
O53—Ag20—S13	126.1 (3)	O50—Ag47—O9	118.0 (4)
O53—Ag20—S14	101.8 (3)	O50—Ag47—S7	123.1 (4)
S13—Ag20—S14	117.04 (15)	O50—Ag47—S8	98.5 (4)
S14—Ag21—S15	148.66 (16)	S7—Ag47—S8	112.09 (13)
O40—Ag22—S15	92.4 (4)	O10—Ag48—S21	105.8 (3)
O40—Ag22—S16	91.4 (3)	O45—Ag48—O10	99.7 (4)
O41—Ag22—O40	118.1 (4)	O45—Ag48—S21	85.8 (3)
O41—Ag22—S15	105.3 (4)	O45—Ag48—S22	147.2 (3)

O41—Ag22—S16	114.8 (3)	S22—Ag48—O10	106.6 (3)
S16—Ag22—S15	131.82 (14)	S22—Ag48—S21	104.97 (14)
O42—Ag23—S16	113.9 (3)	S20—Ag49—O47	106.2 (3)
O42—Ag23—S17	97.0 (3)	S20—Ag49—S22	157.91 (14)
S16—Ag23—S17	141.65 (15)	S22—Ag49—O47	93.7 (3)
S15—Ag24—S17	169.29 (19)	N1—Ag50—S3	108.8 (5)
O23—Ag25—O64	82.3 (4)	N1—Ag50—S4	115.1 (5)
O23—Ag25—S13	129.1 (3)	S4—Ag50—S3	135.92 (17)
O23—Ag25—S15	100.8 (3)		
**SD/Ag50a**			
Ag1—Ag1^{i}	3.026 (7)	Ag6—S6	2.460 (4)
Ag1—Ag2	3.226 (4)	Ag7—Ag8	2.9871 (19)
Ag1—Ag2^{i}	3.009 (3)	Ag7—Ag9	2.859 (2)
Ag1—Ag13	3.194 (4)	Ag7—Ag11	2.9397 (19)
Ag1—O1	2.566 (8)	Ag7—O14	2.242 (15)
Ag1—S1	2.556 (10)	Ag7—S4	2.521 (5)
Ag1—S2	2.487 (13)	Ag7—S6	2.506 (5)
Ag2—Ag3	3.140 (3)	Ag8—Ag9	3.167 (2)
Ag2—Ag10^{ii}	2.902 (3)	Ag8—O2^{iii}	2.356 (9)
Ag2—O8	2.40 (2)	Ag8—O11^{ii}	2.400 (13)
Ag2—O9	2.29 (2)	Ag8—S5	2.567 (6)
Ag2—S1	2.596 (11)	Ag8—S6	2.482 (4)
Ag2—S2^{i}	2.605 (16)	Ag9—Ag12^{iii}	3.016 (2)
Ag3—Ag8^{ii}	3.348 (2)	Ag9—O4^{iii}	2.394 (8)
Ag3—Ag10^{ii}	3.085 (2)	Ag9—S4	2.506 (4)
Ag3—N1	2.36 (2)	Ag9—S5	2.444 (5)
Ag3—O11	2.598 (14)	Ag10—O10^{ii}	2.318 (19)
Ag3—S1	2.628 (10)	Ag10—S2^{iii}	2.152 (12)
Ag3—S5^{ii}	2.562 (6)	Ag10—S5	2.426 (6)
Ag4—Ag5	3.342 (9)	Ag11—Ag12	2.985 (2)
Ag4—Ag6	3.0246 (16)	Ag11—O13	2.353 (14)
Ag4—O2^{i}	2.456 (8)	Ag11—O16	2.56 (3)
Ag4—O12	2.458 (14)	Ag11—S3	2.503 (4)
Ag4—S1	2.541 (10)	Ag11—S4	2.575 (4)
Ag4—S3	2.586 (4)	Ag12—Ag12^{iii}	2.843 (4)
Ag5—Ag5^{ii}	2.594 (19)	Ag12—O5^{i}	2.314 (8)
Ag5—Ag6	3.047 (10)	Ag12—O15	2.21 (3)
Ag5—Ag8^{ii}	2.660 (11)	Ag12—S4	2.914 (4)
Ag5—O6^{i}	2.321 (11)	Ag12—S4^{iii}	2.587 (5)
Ag5—O12	2.413 (18)	Ag13—O4	2.489 (8)
Ag5—S6^{ii}	2.563 (10)	Ag13—O15	2.53 (3)
Ag6—Ag7	2.9655 (19)	Ag13—S2	2.510 (14)
Ag6—Ag11	2.957 (2)	Ag13—S3	2.514 (4)

Ag6—S3	2.456 (4)		
S1—Ag1—O1	127.5 (3)	O2^{iii}—Ag8—S5	92.7 (2)
S2—Ag1—O1	76.8 (4)	O2^{iii}—Ag8—S6	113.9 (2)
O8—Ag2—S1	88.0 (6)	O11^{ii}—Ag8—S5	91.3 (4)
O8—Ag2—S2^{i}	112.9 (6)	O11^{ii}—Ag8—S6	110.3 (3)
O9—Ag2—O8	107.6 (7)	S6—Ag8—S5	143.69 (19)
O9—Ag2—S1	111.3 (5)	O4^{iii}—Ag9—S4	106.62 (19)
O9—Ag2—S2^{i}	103.6 (6)	O4^{iii}—Ag9—S5	96.3 (2)
N1—Ag3—O11	89.2 (7)	S5—Ag9—S4	151.77 (19)
N1—Ag3—S1	115.1 (8)	O10^{ii}—Ag10—S5	108.7 (5)
N1—Ag3—S5^{ii}	104.1 (7)	S2^{iii}—Ag10—O10^{ii}	106.4 (6)
O11—Ag3—S1	105.6 (5)	S2^{iii}—Ag10—S5	144.1 (4)
S5^{ii}—Ag3—O11	87.1 (4)	O13—Ag11—O16	95.3 (9)
S5^{ii}—Ag3—S1	138.6 (3)	O13—Ag11—S3	108.7 (5)
O2^{i}—Ag4—O12	92.5 (5)	O13—Ag11—S4	98.5 (4)
O2^{i}—Ag4—S1	100.2 (3)	O16—Ag11—S4	95.3 (6)
O2^{i}—Ag4—S3	134.41 (19)	S3—Ag11—O16	103.1 (7)
O12—Ag4—S1	117.0 (4)	S3—Ag11—S4	145.22 (14)
O12—Ag4—S3	100.4 (4)	O5^{i}—Ag12—S4	74.8 (2)
S1—Ag4—S3	111.8 (2)	O15—Ag12—O5^{i}	120.0 (7)
O6^{i}—Ag5—O12	104.3 (6)	O15—Ag12—S4	121.9 (7)
O6^{i}—Ag5—S6^{ii}	127.8 (4)	S4^{iii}—Ag12—S4	110.38 (14)
O12—Ag5—S6^{ii}	125.5 (5)	O4—Ag13—O15	76.2 (7)
S3—Ag6—S6	164.96 (14)	O4—Ag13—S2	98.8 (4)
O14—Ag7—S4	105.4 (5)	O4—Ag13—S3	120.94 (18)
O14—Ag7—S6	104.4 (5)	S2—Ag13—O15	106.7 (7)
S6—Ag7—S4	147.80 (13)	S2—Ag13—S3	135.5 (3)
O2^{iii}—Ag8—O11^{ii}	93.5 (4)	S3—Ag13—O15	101.8 (6)
Symmetry codes: (i) −*x*+1/4, −*y*+5/4, *z*; (ii) −*x*+1/4, *y*, −*z*+5/4; (iii) *x*, −*y*+5/4, −*z*+5/4.			
**SD/Ag50b**			
Ag1—Ag3	2.989 (2)	Ag24—S19	2.525 (3)
Ag1—Ag4	3.051 (3)	Ag25—Ag26	2.9889 (15)
Ag1—Ag7	3.157 (3)	Ag25—O56	2.390 (10)
Ag1—S1	2.542 (5)	Ag25—S15	2.470 (4)
Ag1—S24	2.451 (4)	Ag25—S19	2.491 (3)
Ag2—Ag13	3.1427 (17)	Ag26—O3	2.472 (7)
Ag2—Ag27	3.0998 (19)	Ag26—O55	2.416 (9)
Ag2—Ag28	2.826 (2)	Ag26—S15	2.501 (3)
Ag2—Ag29	3.3558 (18)	Ag26—S23	2.535 (3)
Ag2—S13	2.341 (3)	Ag27—Ag28	2.9139 (14)
Ag2—S14	2.400 (4)	Ag27—O59	2.308 (9)
Ag3—Ag4	2.8889 (14)	Ag27—O61	2.384 (10)
Ag3—Ag5	3.2729 (14)	Ag27—S13	2.596 (3)

Ag3—O48	2.306 (11)	Ag27—S23	2.593 (4)
Ag3—O60	2.473 (9)	Ag28—O62	2.297 (11)
Ag3—S20	2.548 (3)	Ag28—S14	2.536 (4)
Ag3—S24	2.620 (4)	Ag28—S23	2.464 (4)
Ag4—Ag8	3.1579 (17)	Ag29—Ag30	3.175 (2)
Ag4—O47	2.465 (12)	Ag29—Ag31	2.8613 (17)
Ag4—S1	2.519 (4)	Ag29—S4	2.474 (3)
Ag4—S20	2.481 (3)	Ag29—S14	2.406 (4)
Ag5—Ag6	3.1725 (15)	Ag30—Ag33	3.1433 (17)
Ag5—Ag12	3.3443 (14)	Ag30—O3	2.283 (8)
Ag5—O60	2.489 (9)	Ag30—O55	2.357 (10)
Ag5—S23	2.448 (3)	Ag30—S5	2.431 (3)
Ag5—S24	2.522 (4)	Ag30—S14	2.680 (4)
Ag6—O7	2.557 (7)	Ag31—Ag32	3.1189 (14)
Ag6—S15	2.477 (3)	Ag31—Ag34	2.8444 (17)
Ag6—S24	2.476 (4)	Ag31—O35	2.372 (17)
Ag7—Ag9	2.9464 (17)	Ag31—S4	2.748 (3)
Ag7—Ag21	2.9500 (16)	Ag31—S5	2.463 (3)
Ag7—O44	2.328 (11)	Ag32—Ag33	3.2385 (12)
Ag7—O45	2.245 (11)	Ag32—Ag34	3.1803 (13)
Ag7—S1	2.482 (4)	Ag32—Ag42	2.9506 (11)
Ag8—Ag9	3.1513 (17)	Ag32—O33	2.365 (7)
Ag8—Ag10	2.9519 (18)	Ag32—S5	2.520 (3)
Ag8—O8	2.320 (7)	Ag32—S18	2.500 (3)
Ag8—O49	2.337 (11)	Ag33—O17	2.581 (6)
Ag8—S1	2.592 (4)	Ag33—O19	2.428 (6)
Ag8—S22	2.487 (3)	Ag33—S5	2.517 (3)
Ag9—Ag20	3.2297 (16)	Ag33—S19	2.460 (3)
Ag9—O43	2.246 (10)	Ag34—Ag35	3.3633 (13)
Ag9—S8	2.523 (3)	Ag34—Ag43	3.2850 (13)
Ag9—S22	2.473 (3)	Ag34—O36	2.330 (16)
Ag10—Ag19	3.2283 (16)	Ag34—S4	2.531 (3)
Ag10—O10	2.563 (7)	Ag34—S18	2.439 (3)
Ag10—O12	2.574 (6)	Ag35—Ag36	3.0908 (13)
Ag10—S7	2.470 (3)	Ag35—O18	2.360 (6)
Ag10—S22	2.486 (3)	Ag35—O37	2.382 (8)
Ag11—Ag12	3.3727 (14)	Ag35—S3	2.538 (3)
Ag11—Ag16	3.0889 (12)	Ag35—S4	2.962 (3)
Ag11—O8	2.467 (7)	Ag36—Ag37	3.1247 (16)
Ag11—O50	2.443 (10)	Ag36—O20	2.526 (6)
Ag11—S9	2.572 (3)	Ag36—O38	2.520 (8)
Ag11—S20	2.516 (3)	Ag36—S3	2.464 (3)
Ag12—Ag13	3.1020 (14)	Ag36—S17	2.462 (3)

Ag12—S13	2.471 (3)	Ag37—Ag38	2.9066 (15)
Ag12—S20	2.464 (3)	Ag37—Ag39	3.1848 (17)
Ag13—Ag14	3.3338 (14)	Ag37—S16	2.441 (3)
Ag13—O2	2.517 (7)	Ag37—S17	2.543 (3)
Ag13—O54	2.478 (15)	Ag38—Ag39	2.9447 (18)
Ag13—S9	2.474 (3)	Ag38—O63	2.308 (14)
Ag13—S13	2.546 (3)	Ag38—S11	2.436 (3)
Ag14—Ag15	3.0072 (14)	Ag38—S17	2.484 (3)
Ag14—Ag29	2.8931 (17)	Ag39—Ag40	3.2201 (15)
Ag14—Ag35	2.8983 (14)	Ag39—Ag41	3.2071 (16)
Ag14—O2	2.514 (7)	Ag39—O29	2.282 (19)
Ag14—O15	2.351 (6)	Ag39—O64	2.52 (3)
Ag14—O54	2.426 (16)	Ag39—S11	2.582 (3)
Ag14—S4	2.516 (3)	Ag39—S16	2.559 (3)
Ag15—Ag16	2.9963 (14)	Ag40—Ag42	3.3375 (12)
Ag15—Ag17	2.9820 (14)	Ag40—Ag43	3.1831 (13)
Ag15—O53	2.547 (16)	Ag40—S12	2.455 (3)
Ag15—S3	2.443 (3)	Ag40—S16	2.470 (3)
Ag15—S9	2.421 (3)	Ag41—Ag47	3.1087 (15)
Ag16—Ag17	2.8687 (12)	Ag41—Ag48	3.3003 (18)
Ag16—O50	2.529 (10)	Ag41—O25	2.535 (7)
Ag16—O51	2.307 (9)	Ag41—S11	2.454 (3)
Ag16—S7	2.554 (3)	Ag41—S21	2.497 (4)
Ag16—S9	2.535 (3)	Ag42—O27	2.448 (6)
Ag17—Ag18	3.0317 (13)	Ag42—O34	2.330 (7)
Ag17—Ag36	3.0056 (13)	Ag42—S12	2.581 (3)
Ag17—O52	2.308 (10)	Ag42—S18	2.525 (3)
Ag17—S3	2.534 (3)	Ag43—O20	2.440 (7)
Ag17—S7	2.574 (3)	Ag43—O37	2.475 (8)
Ag18—Ag36	3.0953 (12)	Ag43—S16	2.541 (3)
Ag18—O23	2.401 (6)	Ag43—S18	2.514 (3)
Ag18—O39	2.521 (10)	Ag44—Ag46	3.0939 (18)
Ag18—S7	2.444 (3)	Ag44—Ag48	2.913 (2)
Ag18—S17	2.495 (3)	Ag44—S6	2.445 (3)
Ag19—Ag20	3.0500 (12)	Ag44—S12	2.430 (3)
Ag19—Ag50	2.9361 (13)	Ag45—Ag49	3.1154 (11)
Ag19—O40	2.285 (8)	Ag45—O27	2.360 (6)
Ag19—S10	2.544 (3)	Ag45—O34	2.498 (8)
Ag19—S22	2.512 (3)	Ag45—S6	2.508 (3)
Ag20—Ag22	3.2099 (11)	Ag45—S19	2.451 (3)
Ag20—Ag47	3.3693 (14)	Ag46—Ag48	3.0631 (19)
Ag20—S8	2.405 (3)	Ag46—Ag49	3.1712 (15)
Ag20—S10	2.393 (3)	Ag46—O32	2.390 (19)

Ag21—Ag22	2.9579 (14)	Ag46—S6	2.516 (3)
Ag21—O7	2.421 (7)	Ag46—S21	2.508 (4)
Ag21—O9	2.425 (6)	Ag47—O26	2.508 (6)
Ag21—O46	2.277 (9)	Ag47—O41	2.515 (7)
Ag21—S8	2.436 (3)	Ag47—S10	2.499 (3)
Ag22—Ag49	2.9877 (12)	Ag47—S21	2.517 (4)
Ag22—O13	2.351 (6)	Ag48—O30	2.405 (15)
Ag22—O41	2.343 (8)	Ag48—O31	2.419 (19)
Ag22—S2	2.513 (3)	Ag48—S12	2.569 (3)
Ag23—Ag24	2.8478 (13)	Ag48—S21	2.565 (4)
Ag23—Ag25	3.3230 (16)	Ag49—O26	2.551 (6)
Ag23—O58	2.386 (8)	Ag49—O42	2.475 (7)
Ag23—S2	2.511 (3)	Ag49—S2	2.482 (3)
Ag23—S15	2.494 (3)	Ag49—S6	2.472 (3)
Ag24—Ag25	3.2777 (15)	Ag50—O23	2.406 (7)
Ag24—Ag45	2.9908 (12)	Ag50—O39	2.322 (9)
Ag24—Ag49	2.9351 (12)	Ag50—S10	2.543 (3)
Ag24—O57	2.305 (7)	Ag50—S11	2.554 (3)
Ag24—S2	2.576 (3)		
S24—Ag1—S1	139.1 (2)	O59—Ag27—S23	109.0 (3)
S13—Ag2—S14	169.68 (15)	O61—Ag27—S13	109.7 (3)
O48—Ag3—O60	121.8 (4)	O61—Ag27—S23	108.4 (3)
O48—Ag3—S20	119.1 (3)	S23—Ag27—S13	124.43 (10)
O48—Ag3—S24	99.0 (4)	O62—Ag28—S14	102.4 (4)
O60—Ag3—S20	92.3 (2)	O62—Ag28—S23	108.0 (4)
O60—Ag3—S24	91.1 (2)	S23—Ag28—S14	148.20 (11)
S20—Ag3—S24	131.96 (10)	S14—Ag29—S4	166.80 (11)
O47—Ag4—S1	105.8 (4)	O3—Ag30—O55	76.7 (3)
O47—Ag4—S20	97.1 (4)	O3—Ag30—S5	130.1 (2)
S20—Ag4—S1	151.08 (11)	O3—Ag30—S14	86.4 (2)
O60—Ag5—S24	93.1 (2)	O55—Ag30—S5	125.5 (3)
S23—Ag5—O60	108.6 (2)	O55—Ag30—S14	100.5 (3)
S23—Ag5—S24	143.53 (11)	S5—Ag30—S14	123.99 (12)
S15—Ag6—O7	126.93 (17)	O35—Ag31—S4	97.0 (4)
S24—Ag6—O7	93.47 (18)	O35—Ag31—S5	121.9 (4)
S24—Ag6—S15	137.30 (11)	S5—Ag31—S4	132.07 (9)
O44—Ag7—S1	105.0 (3)	O33—Ag32—S5	96.2 (2)
O45—Ag7—O44	96.5 (4)	O33—Ag32—S18	115.1 (2)
O45—Ag7—S1	131.9 (4)	S18—Ag32—S5	148.65 (9)
O8—Ag8—O49	101.6 (3)	O19—Ag33—O17	72.6 (2)
O8—Ag8—S1	94.67 (19)	O19—Ag33—S5	83.16 (16)
O8—Ag8—S22	117.9 (2)	O19—Ag33—S19	129.03 (16)
O49—Ag8—S1	94.7 (3)	S5—Ag33—O17	128.99 (16)

O49—Ag8—S22	121.6 (3)	S19—Ag33—O17	79.22 (15)
S22—Ag8—S1	120.86 (12)	S19—Ag33—S5	145.39 (9)
O43—Ag9—S8	109.8 (3)	O36—Ag34—S4	91.8 (4)
O43—Ag9—S22	119.7 (3)	O36—Ag34—S18	118.1 (4)
S22—Ag9—S8	125.81 (10)	S18—Ag34—S4	149.78 (9)
O10—Ag10—O12	69.8 (2)	O18—Ag35—O37	106.2 (3)
S7—Ag10—O10	77.49 (15)	O18—Ag35—S3	136.65 (17)
S7—Ag10—O12	112.67 (16)	O18—Ag35—S4	68.63 (16)
S7—Ag10—S22	146.60 (10)	O37—Ag35—S3	113.0 (2)
S22—Ag10—O10	135.33 (15)	O37—Ag35—S4	95.3 (2)
S22—Ag10—O12	81.08 (16)	S3—Ag35—S4	123.30 (9)
O8—Ag11—S9	131.93 (18)	O38—Ag36—O20	85.9 (2)
O8—Ag11—S20	97.78 (18)	S3—Ag36—O20	101.86 (17)
O50—Ag11—O8	88.1 (3)	S3—Ag36—O38	101.0 (2)
O50—Ag11—S9	100.3 (3)	S17—Ag36—O20	100.37 (17)
O50—Ag11—S20	115.2 (3)	S17—Ag36—O38	97.1 (2)
S20—Ag11—S9	119.72 (11)	S17—Ag36—S3	152.15 (10)
S20—Ag12—S13	154.26 (11)	S16—Ag37—S17	151.48 (11)
O2—Ag13—S13	99.92 (18)	O63—Ag38—S11	119.9 (4)
O54—Ag13—O2	78.2 (4)	O63—Ag38—S17	104.4 (4)
O54—Ag13—S13	101.3 (4)	S11—Ag38—S17	135.23 (10)
S9—Ag13—O2	120.91 (17)	O29—Ag39—O64	98.3 (8)
S9—Ag13—O54	109.1 (4)	O29—Ag39—S11	106.4 (5)
S9—Ag13—S13	132.64 (11)	O29—Ag39—S16	115.9 (5)
O2—Ag14—S4	106.30 (18)	O64—Ag39—S11	109.3 (6)
O15—Ag14—O2	74.0 (2)	O64—Ag39—S16	88.5 (7)
O15—Ag14—O54	122.3 (4)	S16—Ag39—S11	130.96 (10)
O15—Ag14—S4	121.42 (17)	S12—Ag40—S16	149.28 (11)
O54—Ag14—O2	79.2 (4)	S11—Ag41—O25	134.57 (17)
O54—Ag14—S4	114.8 (4)	S11—Ag41—S21	144.89 (11)
S3—Ag15—O53	92.0 (4)	S21—Ag41—O25	76.66 (17)
S9—Ag15—O53	104.9 (4)	O27—Ag42—S12	100.68 (17)
S9—Ag15—S3	162.67 (10)	O27—Ag42—S18	126.84 (16)
O50—Ag16—S7	92.1 (3)	O34—Ag42—O27	77.9 (2)
O50—Ag16—S9	99.1 (2)	O34—Ag42—S12	100.5 (2)
O51—Ag16—O50	102.4 (4)	O34—Ag42—S18	128.7 (2)
O51—Ag16—S7	97.5 (3)	S18—Ag42—S12	114.33 (10)
O51—Ag16—S9	100.8 (3)	O20—Ag43—O37	83.6 (2)
S9—Ag16—S7	155.86 (9)	O20—Ag43—S16	99.73 (17)
O52—Ag17—S3	117.4 (3)	O20—Ag43—S18	128.70 (16)
O52—Ag17—S7	97.8 (3)	O37—Ag43—S16	113.1 (2)
S3—Ag17—S7	142.47 (8)	O37—Ag43—S18	104.6 (2)
O23—Ag18—O39	77.2 (3)	S18—Ag43—S16	121.04 (10)

O23—Ag18—S7	113.70 (17)	S12—Ag44—S6	142.89 (11)
O23—Ag18—S17	87.46 (17)	O27—Ag45—O34	76.3 (2)
S7—Ag18—O39	108.6 (3)	O27—Ag45—S6	90.70 (17)
S7—Ag18—S17	150.88 (10)	O27—Ag45—S19	121.22 (18)
S17—Ag18—O39	95.0 (3)	O34—Ag45—S6	97.6 (2)
O40—Ag19—S10	118.1 (3)	S19—Ag45—O34	108.4 (2)
O40—Ag19—S22	101.0 (3)	S19—Ag45—S6	142.30 (10)
S22—Ag19—S10	137.31 (9)	O32—Ag46—S6	106.1 (4)
S10—Ag20—S8	171.63 (9)	O32—Ag46—S21	102.1 (4)
O7—Ag21—O9	74.5 (2)	S21—Ag46—S6	143.27 (11)
O7—Ag21—S8	116.84 (18)	O26—Ag47—O41	81.9 (2)
O9—Ag21—S8	129.52 (17)	O26—Ag47—S21	94.07 (17)
O46—Ag21—O7	94.1 (3)	O41—Ag47—S21	108.1 (2)
O46—Ag21—O9	96.5 (3)	S10—Ag47—O26	126.17 (16)
O46—Ag21—S8	128.7 (2)	S10—Ag47—O41	103.17 (19)
O13—Ag22—S2	130.53 (17)	S10—Ag47—S21	131.88 (11)
O41—Ag22—O13	109.6 (2)	O30—Ag48—O31	124.0 (5)
O41—Ag22—S2	116.83 (19)	O30—Ag48—S12	90.5 (4)
O58—Ag23—S2	104.2 (2)	O30—Ag48—S21	95.3 (4)
O58—Ag23—S15	98.7 (2)	O31—Ag48—S12	108.8 (5)
S15—Ag23—S2	150.62 (9)	O31—Ag48—S21	105.9 (5)
O57—Ag24—S2	104.1 (2)	S21—Ag48—S12	133.22 (11)
O57—Ag24—S19	107.4 (2)	O42—Ag49—O26	87.9 (2)
S19—Ag24—S2	145.23 (8)	O42—Ag49—S2	101.1 (2)
O56—Ag25—S15	115.7 (3)	S2—Ag49—O26	104.77 (16)
O56—Ag25—S19	97.8 (3)	S6—Ag49—O26	99.40 (16)
S15—Ag25—S19	146.31 (10)	S6—Ag49—O42	96.8 (2)
O3—Ag26—S15	128.84 (18)	S6—Ag49—S2	150.25 (10)
O3—Ag26—S23	99.89 (19)	O23—Ag50—S10	125.34 (16)
O55—Ag26—O3	72.2 (3)	O23—Ag50—S11	104.49 (18)
O55—Ag26—S15	122.2 (3)	O39—Ag50—O23	81.1 (3)
O55—Ag26—S23	96.6 (3)	O39—Ag50—S10	122.8 (3)
S15—Ag26—S23	123.31 (12)	O39—Ag50—S11	105.2 (3)
O59—Ag27—O61	102.5 (4)	S10—Ag50—S11	112.80 (10)
O59—Ag27—S13	100.4 (3)		
**SD/Ag50c**			
Ag1—Ag2	3.1030 (14)	Ag25—S9	2.5298 (16)
Ag1—Ag3	2.9449 (15)	Ag26—Ag27	3.2895 (7)
Ag1—Ag20	3.0297 (12)	Ag26—Ag44	3.1050 (7)
Ag1—S3	2.4591 (17)	Ag26—Ag45	3.1861 (7)
Ag1—S5	2.4599 (19)	Ag26—O22	2.411 (4)
Ag2—Ag7	3.2531 (10)	Ag26—O47	2.369 (5)
Ag2—O28	2.577 (4)	Ag26—S6	2.5839 (15)

Ag2—O40	2.282 (6)	Ag26—S8	2.4939 (16)
Ag2—S3	2.5054 (19)	Ag27—Ag28	3.1455 (7)
Ag2—S24	2.553 (2)	Ag27—Ag44	2.8741 (7)
Ag3—Ag4	3.2563 (8)	Ag27—Ag48	2.8061 (7)
Ag3—Ag40	3.2016 (7)	Ag27—O46	2.310 (5)
Ag3—Ag42	3.0159 (7)	Ag27—S7	2.5752 (16)
Ag3—O23	2.537 (4)	Ag27—S8	2.4245 (17)
Ag3—O51	2.470 (5)	Ag28—Ag29	2.9292 (6)
Ag3—S3	2.4914 (17)	Ag28—Ag48	3.3448 (8)
Ag3—S4	2.5967 (17)	Ag28—O44	2.366 (5)
Ag4—Ag5	2.9853 (8)	Ag28—S8	2.5277 (16)
Ag4—Ag40	3.0957 (7)	Ag28—S13	2.5155 (16)
Ag4—O52	2.305 (5)	Ag29—Ag30	3.2984 (7)
Ag4—O53	2.383 (6)	Ag29—O1	2.449 (4)
Ag4—S2	2.6501 (18)	Ag29—O43	2.321 (5)
Ag4—S3	2.5915 (18)	Ag29—S12	2.5621 (16)
Ag5—Ag6	3.2234 (8)	Ag29—S13	2.5389 (17)
Ag5—O54	2.273 (6)	Ag30—O34	2.320 (5)
Ag5—S2	2.4267 (19)	Ag30—S11	2.4936 (17)
Ag5—S24	2.4663 (18)	Ag30—S12	2.4439 (19)
Ag6—Ag7	3.0480 (9)	Ag31—Ag32	3.0554 (8)
Ag6—Ag8	3.2347 (8)	Ag31—Ag33	3.0968 (8)
Ag6—O26	2.421 (4)	Ag31—O8	2.538 (4)
Ag6—O55	2.294 (6)	Ag31—O64	2.416 (11)
Ag6—S23	2.5627 (17)	Ag31—S12	2.4866 (19)
Ag6—S24	2.5083 (18)	Ag31—S18	2.553 (2)
Ag7—Ag10	2.8823 (9)	Ag32—O10	2.505 (4)
Ag7—Ag18	2.8889 (8)	Ag32—S13	2.4554 (16)
Ag7—S21	2.4181 (16)	Ag32—S18	2.4807 (17)
Ag7—S24	2.4098 (17)	Ag33—Ag34	3.2716 (8)
Ag8—Ag9	3.2728 (7)	Ag33—Ag35	2.8739 (8)
Ag8—O19	2.493 (4)	Ag33—O32	2.337 (6)
Ag8—O66	2.585 (4)	Ag33—O65	2.247 (10)
Ag8—S16	2.5810 (16)	Ag33—S18	2.5917 (18)
Ag8—S23	2.5189 (16)	Ag33—S19	2.6187 (19)
Ag9—Ag10	3.1515 (8)	Ag34—Ag35	3.0158 (8)
Ag9—Ag11	3.0779 (7)	Ag34—Ag49	3.1279 (7)
Ag9—Ag12	3.2807 (8)	Ag34—N2	2.337 (7)
Ag9—O38	2.345 (5)	Ag34—S17	2.5317 (18)
Ag9—S22	2.5304 (17)	Ag34—S18	2.4527 (19)
Ag9—S23	2.5303 (16)	Ag35—O31	2.303 (5)
Ag10—Ag12	2.8729 (8)	Ag35—S17	2.4721 (18)
Ag10—O29	2.385 (5)	Ag35—S19	2.476 (2)

Ag10—S21	2.6479 (17)	Ag36—Ag37	2.8714 (7)
Ag10—S23	2.4947 (17)	Ag36—Ag49	3.1634 (7)
Ag11—O9	2.395 (4)	Ag36—O9	2.355 (4)
Ag11—O37	2.343 (5)	Ag36—O37	2.439 (5)
Ag11—S19	2.5791 (18)	Ag36—S16	2.4709 (17)
Ag11—S22	2.5074 (17)	Ag36—S17	2.4960 (17)
Ag12—Ag13	3.2787 (7)	Ag37—Ag38	2.8689 (7)
Ag12—Ag17	3.3523 (7)	Ag37—Ag49	2.9844 (7)
Ag12—O30	2.341 (5)	Ag37—Ag50	2.9513 (7)
Ag12—S21	2.5334 (16)	Ag37—O57	2.308 (5)
Ag12—S22	2.4429 (17)	Ag37—S14	2.5141 (16)
Ag13—Ag14	3.0415 (7)	Ag37—S16	2.5178 (16)
Ag13—O3	2.468 (4)	Ag38—Ag39	2.9697 (7)
Ag13—S20	2.5379 (16)	Ag38—Ag50	2.9532 (7)
Ag13—S22	2.4736 (17)	Ag38—S15	2.4775 (16)
Ag14—Ag31	3.1872 (8)	Ag38—S16	2.4790 (17)
Ag14—S19	2.453 (2)	Ag39—Ag40	3.2998 (7)
Ag14—S20	2.4724 (18)	Ag39—O26	2.435 (4)
Ag15—Ag16	3.0344 (7)	Ag39—O56	2.442 (5)
Ag15—Ag23	3.0872 (7)	Ag39—S2	2.5113 (17)
Ag15—Ag30	2.9938 (7)	Ag39—S15	2.5951 (16)
Ag15—S11	2.4482 (16)	Ag40—Ag41	3.1029 (7)
Ag15—S20	2.4092 (17)	Ag40—S1	2.4488 (17)
Ag16—Ag30	2.8511 (7)	Ag40—S2	2.4416 (18)
Ag16—Ag31	3.3455 (8)	Ag41—O24	2.444 (4)
Ag16—O33	2.423 (7)	Ag41—O25	2.454 (4)
Ag16—O64	2.435 (12)	Ag41—S1	2.5322 (16)
Ag16—S12	2.6337 (17)	Ag41—S15	2.5441 (16)
Ag16—S20	2.6446 (17)	Ag42—Ag43	2.8582 (7)
Ag17—Ag18	2.8757 (7)	Ag42—Ag45	3.0638 (7)
Ag17—Ag23	2.9570 (7)	Ag42—O50	2.277 (5)
Ag17—O3	2.584 (4)	Ag42—O51	2.427 (5)
Ag17—O5	2.349 (4)	Ag42—S1	2.5979 (17)
Ag17—O36	2.310 (4)	Ag42—S4	2.5707 (16)
Ag17—S10	2.5096 (16)	Ag43—Ag45	3.0373 (7)
Ag18—O14	2.389 (4)	Ag43—O49	2.309 (4)
Ag18—O28	2.518 (4)	Ag43—S1	2.4843 (17)
Ag18—O39	2.243 (5)	Ag43—S6	2.4443 (15)
Ag18—S21	2.5215 (16)	Ag44—Ag46	2.9504 (7)
Ag19—Ag21	3.0378 (7)	Ag44—O24	2.364 (4)
Ag19—Ag22	2.9041 (7)	Ag44—S6	2.4585 (15)
Ag19—O39	2.565 (6)	Ag44—S7	2.4506 (15)
Ag19—O42	2.367 (6)	Ag45—N1	2.346 (6)

Ag19—S5	2.5254 (16)	Ag45—O47	2.596 (5)
Ag19—S10	2.5434 (16)	Ag45—S4	2.4744 (17)
Ag20—Ag21	3.0891 (7)	Ag45—S6	2.5927 (15)
Ag20—O22	2.398 (4)	Ag46—Ag47	2.7936 (7)
Ag20—O48	2.517 (5)	Ag46—Ag50	3.0557 (7)
Ag20—S4	2.5185 (15)	Ag46—O20	2.377 (4)
Ag20—S5	2.5460 (16)	Ag46—O25	2.293 (4)
Ag21—Ag22	2.9094 (7)	Ag46—S7	2.6795 (16)
Ag21—Ag25	3.1689 (8)	Ag46—S14	2.7526 (15)
Ag21—S5	2.4245 (17)	Ag47—Ag48	3.2884 (7)
Ag21—S9	2.4357 (16)	Ag47—Ag49	2.8809 (7)
Ag22—Ag23	2.8606 (7)	Ag47—O10	2.557 (4)
Ag22—Ag24	2.9601 (7)	Ag47—O18	2.387 (4)
Ag22—O41	2.262 (5)	Ag47—O62	2.253 (4)
Ag22—S9	2.5717 (16)	Ag47—S7	2.9176 (15)
Ag22—S10	2.5863 (16)	Ag47—S14	2.5577 (16)
Ag23—Ag24	3.1028 (7)	Ag48—O45	2.322 (5)
Ag23—O35	2.467 (4)	Ag48—S7	2.5869 (15)
Ag23—S10	2.4805 (16)	Ag48—S13	2.4745 (17)
Ag23—S11	2.4722 (16)	Ag49—O10	2.507 (4)
Ag24—O1	2.378 (4)	Ag49—O61	2.518 (5)
Ag24—O43	2.458 (5)	Ag49—S14	2.5477 (16)
Ag24—S9	2.4438 (15)	Ag49—S17	2.4710 (17)
Ag24—S11	2.5115 (15)	Ag50—O58	2.332 (5)
Ag25—Ag26	3.1376 (8)	Ag50—O63	2.421 (5)
Ag25—O15	2.467 (4)	Ag50—S14	2.6157 (16)
Ag25—S8	2.4567 (17)	Ag50—S15	2.5389 (16)
S3—Ag1—S5	141.39 (6)	O46—Ag27—S8	113.42 (14)
O40—Ag2—O28	94.28 (17)	S8—Ag27—S7	147.18 (5)
O40—Ag2—S3	123.63 (18)	O44—Ag28—S8	93.87 (13)
O40—Ag2—S24	105.40 (18)	O44—Ag28—S13	118.96 (13)
S3—Ag2—O28	100.81 (10)	S13—Ag28—S8	146.14 (6)
S3—Ag2—S24	129.77 (8)	O1—Ag29—S12	104.27 (10)
S24—Ag2—O28	84.49 (10)	O1—Ag29—S13	131.76 (10)
O23—Ag3—S4	76.63 (10)	O43—Ag29—O1	75.55 (15)
O51—Ag3—O23	112.27 (14)	O43—Ag29—S12	105.10 (14)
O51—Ag3—S3	108.81 (12)	O43—Ag29—S13	124.94 (13)
O51—Ag3—S4	92.75 (12)	S13—Ag29—S12	109.48 (6)
S3—Ag3—O23	121.46 (10)	O34—Ag30—S11	94.48 (15)
S3—Ag3—S4	140.20 (6)	O34—Ag30—S12	118.90 (16)
O52—Ag4—O53	98.9 (2)	S12—Ag30—S11	145.37 (6)
O52—Ag4—S2	106.06 (14)	O8—Ag31—S18	76.56 (10)
O52—Ag4—S3	117.73 (14)	O64—Ag31—O8	107.6 (3)

O53—Ag4—S2	110.53 (15)	O64—Ag31—S12	91.3 (3)
O53—Ag4—S3	97.97 (16)	O64—Ag31—S18	114.8 (3)
S3—Ag4—S2	122.50 (5)	S12—Ag31—O8	126.97 (10)
O54—Ag5—S2	110.28 (18)	S12—Ag31—S18	138.95 (6)
O54—Ag5—S24	106.71 (18)	S13—Ag32—O10	121.37 (10)
S2—Ag5—S24	142.48 (6)	S13—Ag32—S18	141.52 (6)
O26—Ag6—S23	104.66 (10)	S18—Ag32—O10	96.13 (10)
O26—Ag6—S24	94.00 (11)	O32—Ag33—S18	104.25 (17)
O55—Ag6—O26	109.99 (17)	O32—Ag33—S19	111.81 (17)
O55—Ag6—S23	101.75 (15)	O65—Ag33—O32	85.4 (3)
O55—Ag6—S24	115.30 (15)	O65—Ag33—S18	115.2 (3)
S24—Ag6—S23	129.49 (6)	O65—Ag33—S19	105.4 (3)
S24—Ag7—S21	169.36 (6)	S18—Ag33—S19	127.02 (5)
O19—Ag8—O66	69.75 (12)	N2—Ag34—S17	102.8 (2)
O19—Ag8—S16	74.78 (10)	N2—Ag34—S18	116.4 (2)
O19—Ag8—S23	141.81 (10)	S18—Ag34—S17	138.76 (6)
S16—Ag8—O66	107.67 (10)	O31—Ag35—S17	111.16 (17)
S23—Ag8—O66	86.72 (9)	O31—Ag35—S19	113.42 (18)
S23—Ag8—S16	142.74 (5)	S17—Ag35—S19	134.88 (6)
O38—Ag9—S22	108.08 (14)	O9—Ag36—O37	79.94 (15)
O38—Ag9—S23	103.14 (14)	O9—Ag36—S16	113.75 (10)
S23—Ag9—S22	147.71 (6)	O9—Ag36—S17	93.09 (10)
O29—Ag10—S21	106.32 (13)	O37—Ag36—S16	102.58 (13)
O29—Ag10—S23	122.40 (13)	O37—Ag36—S17	101.99 (13)
S23—Ag10—S21	126.30 (5)	S16—Ag36—S17	146.23 (6)
O9—Ag11—S19	99.75 (10)	O57—Ag37—S14	95.91 (14)
O9—Ag11—S22	129.91 (10)	O57—Ag37—S16	112.99 (14)
O37—Ag11—O9	81.11 (15)	S14—Ag37—S16	148.95 (5)
O37—Ag11—S19	99.60 (14)	S15—Ag38—S16	163.43 (5)
O37—Ag11—S22	124.59 (13)	O26—Ag39—O56	87.02 (16)
S22—Ag11—S19	114.33 (6)	O26—Ag39—S2	99.92 (10)
O30—Ag12—S21	95.74 (14)	O26—Ag39—S15	132.10 (10)
O30—Ag12—S22	112.55 (14)	O56—Ag39—S2	116.20 (12)
S22—Ag12—S21	148.67 (6)	O56—Ag39—S15	104.91 (13)
O3—Ag13—S20	94.74 (10)	S2—Ag39—S15	114.46 (6)
O3—Ag13—S22	131.92 (10)	S2—Ag40—S1	147.39 (6)
S22—Ag13—S20	130.34 (5)	O24—Ag41—O25	79.36 (14)
S19—Ag14—S20	148.96 (6)	O24—Ag41—S1	102.99 (10)
S20—Ag15—S11	161.97 (6)	O24—Ag41—S15	118.33 (10)
O33—Ag16—O64	137.2 (3)	O25—Ag41—S1	119.04 (11)
O33—Ag16—S12	110.43 (16)	O25—Ag41—S15	102.58 (12)
O33—Ag16—S20	106.49 (16)	S1—Ag41—S15	125.79 (5)
O64—Ag16—S12	87.5 (3)	O50—Ag42—O51	113.59 (17)

O64—Ag16—S20	90.6 (3)	O50—Ag42—S1	112.02 (13)
S12—Ag16—S20	126.67 (5)	O50—Ag42—S4	111.50 (13)
O5—Ag17—O3	71.68 (13)	O51—Ag42—S1	95.33 (13)
O5—Ag17—S10	136.23 (10)	O51—Ag42—S4	94.40 (12)
O36—Ag17—O3	82.33 (14)	S4—Ag42—S1	126.56 (5)
O36—Ag17—O5	103.11 (15)	O49—Ag43—S1	105.74 (13)
O36—Ag17—S10	119.75 (12)	O49—Ag43—S6	112.19 (13)
S10—Ag17—O3	103.93 (10)	S6—Ag43—S1	140.44 (5)
O14—Ag18—O28	72.43 (13)	O24—Ag44—S6	89.00 (10)
O14—Ag18—S21	125.39 (10)	O24—Ag44—S7	123.04 (10)
O28—Ag18—S21	105.76 (10)	S7—Ag44—S6	147.90 (5)
O39—Ag18—O14	101.21 (18)	N1—Ag45—O47	88.57 (18)
O39—Ag18—O28	102.00 (19)	N1—Ag45—S4	127.04 (15)
O39—Ag18—S21	130.91 (15)	N1—Ag45—S6	96.08 (15)
O42—Ag19—O39	120.20 (19)	S4—Ag45—O47	99.78 (12)
O42—Ag19—S5	111.66 (15)	S4—Ag45—S6	135.22 (5)
O42—Ag19—S10	99.42 (15)	S6—Ag45—O47	91.87 (12)
S5—Ag19—O39	92.03 (14)	O20—Ag46—S7	115.72 (10)
S5—Ag19—S10	142.18 (5)	O20—Ag46—S14	76.51 (10)
S10—Ag19—O39	90.36 (13)	O25—Ag46—O20	111.40 (14)
O22—Ag20—O48	82.27 (15)	O25—Ag46—S7	108.86 (12)
O22—Ag20—S4	95.68 (10)	O25—Ag46—S14	127.85 (13)
O22—Ag20—S5	134.63 (10)	S7—Ag46—S14	112.96 (5)
O48—Ag20—S4	106.83 (11)	O10—Ag47—S7	140.69 (10)
O48—Ag20—S5	100.77 (12)	O10—Ag47—S14	101.94 (10)
S4—Ag20—S5	125.35 (5)	O18—Ag47—O10	72.61 (13)
S5—Ag21—S9	166.44 (6)	O18—Ag47—S7	70.60 (10)
O41—Ag22—S9	105.95 (15)	O18—Ag47—S14	128.37 (10)
O41—Ag22—S10	106.13 (15)	O62—Ag47—O10	87.41 (16)
S9—Ag22—S10	145.29 (5)	O62—Ag47—O18	109.21 (16)
O35—Ag23—S10	99.77 (12)	O62—Ag47—S7	91.90 (13)
O35—Ag23—S11	96.54 (12)	O62—Ag47—S14	121.98 (13)
S11—Ag23—S10	155.97 (6)	S14—Ag47—S7	111.44 (5)
O1—Ag24—O43	74.36 (14)	O45—Ag48—S7	101.76 (13)
O1—Ag24—S9	118.45 (10)	O45—Ag48—S13	109.65 (14)
O1—Ag24—S11	84.91 (10)	S13—Ag48—S7	142.79 (5)
O43—Ag24—S11	96.68 (13)	O10—Ag49—O61	89.07 (14)
S9—Ag24—O43	108.70 (13)	O10—Ag49—S14	103.62 (10)
S9—Ag24—S11	148.97 (6)	O61—Ag49—S14	101.27 (12)
O15—Ag25—S9	80.81 (10)	S17—Ag49—O10	103.81 (10)
S8—Ag25—O15	136.51 (10)	S17—Ag49—O61	101.95 (12)
S8—Ag25—S9	139.48 (6)	S17—Ag49—S14	144.06 (6)
O22—Ag26—S6	94.03 (10)	O58—Ag50—O63	97.4 (2)

O22—Ag26—S8	114.66 (10)	O58—Ag50—S14	105.58 (14)
O47—Ag26—O22	99.51 (15)	O58—Ag50—S15	107.59 (15)
O47—Ag26—S6	97.57 (12)	O63—Ag50—S14	92.76 (13)
O47—Ag26—S8	109.51 (12)	O63—Ag50—S15	107.30 (12)
S8—Ag26—S6	135.44 (5)	S15—Ag50—S14	138.20 (5)
O46—Ag27—S7	99.21 (14)		

Supplementary Methods.

Materials and reagents

The (^{i}PrSAg)$_n$[1] and (^{n}Bu$_4$N)$_4$[α-Mo$_8$O$_{26}$][2] were prepared by following the reported procedures. All chemicals and solvents used in the syntheses were of analytical grade and used without further purification. PhCOOAg was purchased from Nanjing luxury Catalytic materials Co., Ltd. ^{i}PrSH (Adamas-beta®) was purchased from Shanghai Titan Scientific Co., Ltd.

Infrared spectrum

Infrared spectra were recorded on a PerkinElmer Spectrum Two in the frequency range of 4000-500 cm^{-1}.

Elemental analysis

The elemental analyses (C, H, N contents) were determined on a Vario EL III analyzer.

Powder X-ray diffraction

Powder X-ray diffraction (PXRD) data were collected on a Philips X'Pert Pro MPD X-ray diffractometer with CuKα radiation equipped with an X'Celerator detector.

UV/Vis absorption spectra

The UV/Vis absorption spectra were performed on UV−Vis spectrophotometer (Evolution 220, ISA-220 accessory, Thermo Scientific) using a built-in 10 mm silicon photodiode with a 60 mm Spectralon sphere.

Elemental mapping images

Morphology of the samples and elemental composition analyses were measured using an SU-8010 field emission scanning electron microscope (FESEM; Hitachi Ltd., Tokyo, Japan) equipped with an Oxford-Horiba Inca XMax50 energy dispersive X-ray spectroscopy (EDS) attachment (Oxford Instruments Analytical, High Wycombe, England).

Thermogravimetic analysis (TGA)

Thermogravimetic analysis (TGA) was done in a TA SDT Q600 thermal analyzer at

a heating rate of 20°C/min under N_2 atmosphere (200 mL/min) from 20 to 800 °C.

Mass spectra

Mass spectra were recorded on an Agilent 6224 (Agilent Technologies, USA) ESI-TOF-MS spectrometer. Sample solutions are infused by a syringe pump at 240 μL/h. Data were acquired using the following settings: electrospray ionization in positive mode, capillary voltage was set at 3.5 kV (-) and fragmentor at 200 V. The nebulizer was set to 15 psi and the nitrogen drying gas was set to a flow rate of 4 L/min. Drying gas temperature was maintained at 150 ºC. The data analyses of mass spectra were performed based on the isotope distribution patterns using Agilent MassHunter Workstation Data acquisition software (Version B.05.00). The reported *m/z* values represent monoisotopic mass of the most abundant peak within the isotope pattern.

Luminescence measurement

Temperature-dependent photoluminescence measurements were carried out in an Edinburgh spectrofluorimeter (F920S) coupled with an Optistat DN cryostat (Oxford Instruments), and the ITC temperature controller and a pressure gauge were used to realize the variable-temperature measurement in the range of 93-293 K. Spectra were collected at different temperatures after a 3 min homoiothermy. Time-resolved photoluminescence lifetime measurements were performed on the same instrument by using a time-correlated single-photon counting technique.

Supplementary Note 1.

Single crystals of **SD/Ag44** and **SD/Ag50** with appropriate dimensions were chosen under an optical microscope and quickly coated with high vacuum grease (Dow Corning Corporation) to prevent decomposition. Intensity data and cell parameters were recorded at 100 K and 123 K on a Bruker Apex II single crystal diffractometer, employing a Mo K_α radiation (λ = 0.71073 Å) and a CCD area detector. The raw frame data were processed using SAINT and SADABS to yield the reflection data file.[3] Single-crystal X-ray diffraction data of **SD/Ag50a-SD/Ag50c** were collected on a Rigaku Oxford Diffraction XtaLAB Synergy diffractometer equipped with a HyPix-6000HE area detector at 100 K using Mo Kα (λ = 0.71073 Å) from PhotonJet micro-focus X-ray source. These structures were solved using the charge-flipping algorithm, as implemented in the program *SUPERFLIP*[4] and refined by full-matrix least-squares techniques against F_o^2 using the SHELXL program[5] through the OLEX2 interface.[6] Hydrogen atoms at carbon were placed in calculated positions and refined isotropically by using a riding model. Appropriate restraints or constraints were applied to the geometry and the atomic displacement parameters of the atoms in the cluster. All structures were examined using the Addsym subroutine of PLATON[7] to ensure that no additional symmetry could be applied to the models. Pertinent crystallographic data collection and refinement parameters are collated in Supplementary Table 1. Selected bond lengths and angles are collated in Supplementary Table 7.

Supplementary References

1) Wang, Z. *et al.* Johnson solids: anion-templated silver thiolate clusters capped by sulfonate. *Chem-Eur J* **24**, 1640-1650 (2018).

2) Klemperer, W. G. Tetrabutylammonium isopolyoxometalates. *Inorg. Synth*, **27**, 74-85 (1992).

3) Bruker AXS, *APEX2, V2008.6; SADABS V2008/1; SAINT V7.60A; SHELXTL V6.14*; Bruker AXS Inc.: Madison, Wisconsin, USA, **2008**.

4) Palatinus, L. & Chapuis, G. Superflip - a computer program for the solution of crystal structures by charge flipping in arbitrary dimensions. *J Appl Crystallogr* **40**, 786-790, (2007).

5) Sheldrick, G. M. Crystal structure refinement with Shelxl. *Acta Crystallographica Section C-Structural Chemistry* **71**, 3-8, (2015).

6) Dolomanov, O. V., Bourhis, L. J., Gildea, R. J., Howard, J. A. K. & Puschmann, H. Olex2: a complete structure solution, refinement and analysis program. *J Appl Crystallogr* **42**, 339-341, (2009).

7) Spek, A. L. Structure validation in chemical crystallography. *Acta Crystallogr D* **65**, 148-155, (2009).

8) Li, X.-Y. *et al.* Atom-precise polyoxometalate-Ag_2S core-shell nanoparticles. *Chem-Asian J* **10**, 1295-1298 (2015).

9) Gao, G.-G., Cheng, P.-S. & Mak, T. C. W. Acid-induced surface functionalization of polyoxometalate by enclosure in a polyhedral silver-alkynyl cage. *J. Am. Chem. Soc.* **131**, 18257-18259 (2009).

10) Qiao, J., Shi, K. & Wang, Q.-M. A giant silver alkynyl cage with sixty silver(I) ions clustered around polyoxometalate templates. *Angew. Chem., Int. Ed.* **49**, 1765-1767 (2010).

11) Li, X.-Y. *et al.* Anion-templated nanosized silver clusters protected by mixed thiolate and diphosphine. *Nanoscale* **9**, 3601-3608 (2017).

12) Wang, J.-P., Du, X.-D. & Niu, J.-Y. A novel 1D organic-inorganic hybrid based on alternating heteropolyanions $[GeMo_{12}O_{40}]^{4-}$ and isopolyanions $[Mo_6O_{22}]^{8-}$. *J.*

Solid. State. Chem. **179**, 3260-3264 (2006).

13) Dai, L., Wang, E., You, W. & Zhang, Z. Synthesis and electrochemical properties of a new 1D organic-inorganic hybrid compound based on Keggin-type heteropolyanions and isopolyanions decorated by transition metal fragments. *J. Clust. Sci.* **19**, 511-519 (2008).

14) Su, Z.-H., Zhou, B.-B., Zhao, Z.-F. & Zhang, X. A novel 1D chain compound constructed from copper-complex fragments-substituted dilacunary beta-octamolybdate units and saturated beta-octamolybdate clusters. *Inorg Chem Commun* **11**, 334-337 (2008).

15) Li, Y.-W. *et al.* Two unprecedented POM-based inorganic organic hybrids with concomitant heteropolytungstate and molybdate. *Inorg Chem* **56**, 2481-2489 (2017).
